# Phase Separation‐Mediated SRF/P54nrb Transcription Complex Shapes the Vasculature Microenvironment via Upregulating OLFML3 in Glioblastoma

**DOI:** 10.1002/mco2.70759

**Published:** 2026-05-23

**Authors:** Zetao Chen, Yujie Zhang, Liwei Hao, Chenya Feng, Chuangyuan Wang, Tianjing Ai, Peiqian Hu, Jingsen Ji, Shengsong Fu, Taoliang Chen, Fabing Zhang, Liang Zhao, Yiquan Ke

**Affiliations:** ^1^ The National Key Clinical Specialty The Engineering Technology Research Center of Education Ministry of China Guangdong Provincial Key Laboratory on Brain Function Repair and Regeneration Department of Neurosurgery Zhujiang Hospital Southern Medical University Guangzhou China; ^2^ Department of Pathology Nanfang Hospital Southern Medical University Guangzhou China; ^3^ Department of Pathology & Guangdong Province Key Laboratory of Molecular Tumor Pathology School of Basic Medical Sciences Southern Medical University Guangzhou China; ^4^ Department of Information Technology School of Biomedical Engineering Southern Medical University Guangzhou China; ^5^ Department of Radiology Nanfang Hospital Southern Medical University Guangzhou China; ^6^ Department of Radiology Zhujiang Hospital Southern Medical University Guangzhou China; ^7^ Department of Pathology Shunde Hospital Southern Medical University (The First People's Hospital of Shunde) Foshan China; ^8^ Department of Neurosurgery School of Medicine the Sixth Affiliated Hospital of South China University of Technology (Nanhai District People's Hospital of Foshan) Foshan China

**Keywords:** glioblastoma, microenvironment, OLFML3, P54nrb, phase separation, SRF

## Abstract

Antiangiogenic therapy remains a challenging issue in the treatment of glioblastoma (GBM). Effective therapies are in urgent need to improve prognosis of GBM patients. The high heterogeneity of GBM is both the cause of its unavoidable therapeutic resistance and the result of extensive genomic dysregulation, of which abnormal transcription factor (TF) networks are recognized to be the culprit. Herein, based on the heterogeneity of GBM, we identified the key TF serum response factor (SRF), which is closely associated with the formation of the GBM vascular microenvironment, from a large‐scale dataset. Mechanistically, the 133–240aa region of SRF binds to the transcriptional cofactor P54nrb and undergoes phase separation to form a transcription complex, which upregulates OLFML3 by binding to its enhancer and promoter regions. The secreted extracellular matrix (ECM) glycoprotein OLFML3 activates endothelial cells (ECs) by degrading and remodeling the ECM, releasing proangiogenic factors, promoting intercellular adhesion, and directly acting on ECs to promote angiogenesis. The hyperplasia vasculature, in turn, promotes the infiltration of M2‐polarized macrophages, leading to the formation of an immunosuppressive microenvironment. This study comprehensively elucidates the pivotal role of SRF in GBM angiogenesis. Targeting the SRF/P54nrb/OLFML3 axis holds promise for developing novel antiangiogenic strategies to improve GBM treatment outcomes.

## Graphical abstract

We identified SRF as the key TF significantly associated with the malignant phenotype and poor prognosis of GBM. SRF forms a transcriptional complex with P54nrb via phase separation to upregulate the expression of OLFML3. OLFML3 activates ECs and promotes angiogenesis by degrading ECM, releasing proangiogenic factors, enhancing cell–cell adhesion, and directly acting on ECs. The excessive tumor vasculature further promotes the infiltration of M2‐polarized macrophages, leading to the formation of an immunosuppressive microenvironment.

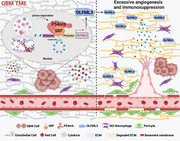

## 1 Introduction

Gliomas are the most common malignant tumors of the central nervous system. Current treatments primarily involve surgical resection followed by adjuvant radiotherapy, chemotherapy, targeted therapy, or immunotherapy. However, the prognosis for patients remains poor, particularly for glioblastoma (GBM), which has high recurrence and mortality rates [1]. Due to the highly vascularized nature of the tumor, antiangiogenic therapy is considered a promising approach for GBM patients. Bevacizumab (a VEGF‐A monoclonal antibody) is the only antiangiogenic drug approved by the United States Food and Drug Administration for GBM treatment. However, clinical practices have shown that bevacizumab can only extend the progression‐free survival (PFS) period without affecting overall survival (OS) [2]. Therefore, it is of great clinical significance to further explore the regulatory mechanisms of GBM angiogenesis and to identify feasible antiangiogenic therapeutic targets to improve treatment outcomes.

The development and progression of tumors are accompanied by extensive dysregulation of the genome, a mechanism precisely regulated by multiple factors. Transcription factors (TFs) are both the final link and the most critical component in this process. There are over 1600 TFs among the 24,000 protein‐coding genes in human cells [3, 4]. They decode the genome by recruiting complex transcription mechanisms to regulate gene expression and play pivotal roles in various physiological [5] and pathological processes [6], such as cell growth, differentiation, metabolism, and tumor progression. As a key part of the molecular signaling network in eukaryotic cells, the proper functioning of TFs is crucial. Numerous studies have shown that in various malignancies, TFs activity is altered through direct mechanisms such as chromosomal translocation, gene amplification or deletion, expression changes, and indirect changes through noncoding DNA mutations that affect TFs binding [7, 8]. A typical example is the tumor suppressor P53, which mutates in over 50% of tumor tissues, thereby losing its original tumor‐suppressing function and being closely associated with the poor prognosis of tumor patients [9]. Since almost all signaling pathways ultimately converge at TFs to exert downstream effects, the prevailing view is that directly targeting abnormal TFs is more specific than targeting other components in the signaling network. This approach is expected to minimize the impact on normal cellular physiological functions and reduce treatment side effects. Although promising TFs such as POU3F2, SOX2, SALL2, and OLIG2 have been identified in GBM [10], these studies remain at the basic research stage, and the development of related targeted drugs is lagging. Therefore, there is a great need to screen for more efficient and feasible new targets.

Serum response factor (SRF) is a highly conserved and ubiquitously expressed TF that binds to CArG‐box cis‐elements on gene promoters, with the aid of various transcriptional cofactors. This binding regulates the expression of downstream target genes and plays a crucial role in numerous physiological processes, such as vascular smooth muscle cell differentiation [11]. In recent years, the roles of SRF in malignancies have garnered increasing attention and research. It has been confirmed to be abnormally expressed in various malignancies and is associated with poor prognosis. For instance, SRF can bind to the promoter region of MYH9 and upregulate its expression, thereby enhancing the migration and invasion capabilities of gastric cancer cells [12]. Additionally, the SRF/MCM7 complex‐mediated expression of downstream target genes significantly promotes liver cancer metastasis and tumor stemness [13]. Furthermore, nucleosome‐mediated sequestration of G‐actin activates the transcriptional activity of the SRF/MRTF complex, thereby promoting the invasive capability of tumor cells [14]. However, its function and mechanisms in GBM remain largely unknown, and no relevant reports have been found.

In this study, using large‐sample multicenter clinical data, we identified SRF as the key TF, which is significantly associated with the malignant phenotype and poor prognosis of GBM. Subsequent in vivo and in vitro experiments demonstrated that SRF forms a transcriptional complex with P54nrb via phase separation to upregulate the expression of OLFML3. OLFML3 activates endothelial cells (ECs) and promotes angiogenesis by degrading the ECM, releasing proangiogenic factors, enhancing cell–cell adhesion, and directly acting on ECs. The excessive tumor vasculature further promotes the infiltration of M2‐polarized macrophages, leading to the formation of an immunosuppressive microenvironment. These findings provide new insights and strategies for developing novel targets of GBM TFs.

## 2 Result

### 2.1 SRF Is a Key TF Mediating the Malignant Phenotype and Poor Prognosis of GBM

**FIGURE 1:**
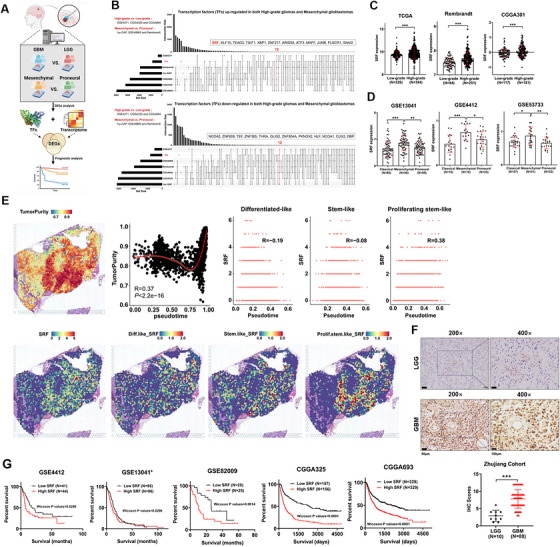
SRF is a key transcription factor mediating the malignant phenotype and poor prognosis of GBM. (A) Schematic representation of the screening process. (B) Upset diagram illustrates the overlap of differential expressed genes (DEGs) between GBM versus LGG and mesenchymal versus proneural in indicated glioma datasets. (C) Scatter diagram represents SRF expression level of GBM and LGG tissues in TCGA, Rembrandt, and CGGA301 datasets. (D) Scatter diagram represents SRF expression level of classical, mesenchymal, and proneural subtypes in the GSE13041, GSE4412, and GSE53733 datasets. (E) Spatial mapping of SRF expression in three GBM subtypes from scRNA‐seq data (Zenodo.496364). Spatial transcriptome data are available in GSE194329. The correlation between SRF expression and pseudotime in each subtype was analyzed separately. (F) IHC results of SRF in LGG and GBM tissues from Zhujiang Hospital. (G) Survival curves for GBM patients with low SRF expression versus high SRF expression through analyzing data from the GSE4412, GSE13041, GSE82009, CGGA325, and CGGA693 databases.

The malignant phenotypes of gliomas comprise of proliferation, invasion, angiogenesis, treatment resistance, and so on. High‐grade glioma (HGG) cells exhibit a higher degree of malignancy in their biological behavior compared with LGG cells. The malignancy degree of GBM, the highest grade of HGG, also varies significantly across different subtypes, reflecting its high heterogeneity. Compelling evidence indicates that GBM cells can be categorized into mesenchymal, classical, and proneural subtypes based on the gene expression profiles [15]. The mesenchymal subtype displays the highest malignancy and poorest prognosis, whereas the proneural subtype exhibits the lowest malignancy and best prognosis. To identify the crucial TFs that mediate the malignant phenotypes of gliomas (especially GBM), we initiated our study by focusing on gene expression profile differences among glioma grades and GBM subtypes. We collected extensive clinical data and gene expression profiles from multiple large‐scale datasets, categorizing the samples into GBM/LGG or mesenchymal/proneural groups. The limma R package was employed to analyze the differentially expressed genes (DEGs) between the two groups. Specifically, the GSE4271, CGGA325, and CGGA694 datasets were utilized to investigate the gene expression disparities between GBM and LGG, while the GSE48865, Ivy‐GAP, and Rembrandt datasets were used to assess differences between the mesenchymal and proneural subtypes (Figure S1A–C). The identified DEGs, including the upregulated and downregulated ones, were further intersected with a set of TFs. Subsequently, we performed univariate and multivariate Cox proportional hazards model analyses on the clinicopathological variables and the identified 24 candidate TFs within the CGGA cohorts (Figure 1A). This analysis revealed that 12 TFs were upregulated and 12 TFs were downregulated in both the GBM and mesenchymal subtypes compared with the LGG and proneural subtypes (Figure 1B). Further survival analysis indicated that SRF, XBP1, DBP, SNAI2, and OLIG2 in the CGGA325 cohort (Table S3), as well as TEAD3, JUNB, SRF, ARID5, and APKNOX in the CGGA693 cohort (Table 1), were independent prognostic factors, with SRF being the sole common element. These results underscored the significant research value of SRF. Consequently, we selected SRF as the object for subsequent research.

**TABLE 1:** Univariate and multivariate cox proportional hazards analysis of clinicopathological variables and 24 candidate TFs based on overall survival (OS) in the CGGA693 cohort.

	Univariate analysis				Multivariate analysis			
OS variables	HR	L95CI	H95CI	*p* value	HR	L95CI	H95CI	*p* value
Age	1.026333	1.017745	1.034994	1.34E‐09	1.018377	1.007764	1.029102	0.000657
Gender	1.060913	0.868091	1.296565	0.563427	—	—	—	—
WHO Grade	2.667433	2.303702	3.088594	2.63E‐39	1.764144	1.414904	2.199585	4.57E‐07
PRS_type	1.477173	1.336107	1.633132	2.57E‐14	1.748085	1.515178	2.016795	1.92E‐14
IDH_mutation	0.323321	0.262361	0.398445	3.23E‐26	0.509362	0.346531	0.748706	0.000598
1p19q_codeletion	0.26791	0.193164	0.371579	2.98E‐15	0.359721	0.224814	0.575583	2.02E‐05
MGMTp_methylation	0.795247	0.638608	0.990307	0.040659	1.059672	0.812077	1.382758	0.669473
SRF	1.042571	1.029751	1.05555	4.00E‐11	1.085994	1.047738	1.125646	6.53E‐06*
XBP1	1.016523	1.011592	1.021478	3.97E‐11	0.97523	0.962834	0.987785	0.000122*
DBP	0.988701	0.985067	0.99235	1.48E‐09	0.98541	0.977689	0.993191	0.00025*
SNAI2	1.07221	1.05551	1.089175	3.17E‐18	1.051179	1.016568	1.086968	0.003478*
OLIG2	0.997326	0.99639	0.998262	2.27E‐08	0.99782	0.996253	0.99939	0.006501*
KLF10	1.043643	1.029338	1.058146	1.31E‐09	0.970153	0.94118	1.000018	0.050136
MAFF	1.024047	1.016225	1.03193	1.25E‐09	0.98148	0.962244	1.0011	0.064162
TEAD3	1.164552	1.13306	1.19692	1.27E‐27	0.940485	0.880253	1.004839	0.069215
TGIF1	1.035618	1.029798	1.04147	4.40E‐34	1.011764	0.998574	1.025128	0.08066
ZNF365	0.980509	0.961924	0.999454	0.043809	0.965325	0.923632	1.0089	0.1172
PLSCR1	1.019841	1.015392	1.024311	1.29E‐18	0.991806	0.981578	1.002141	0.119789
JUNB	1.00176	1.001188	1.002332	1.54E‐09	0.998909	0.997458	1.000361	0.140785
ZNF804A	0.884977	0.800702	0.978122	0.016702	0.917009	0.795381	1.057234	0.23273
THRA	0.995306	0.994025	0.996589	8.21E‐13	0.999164	0.996549	1.001786	0.531572
PKNOX2	0.959935	0.939592	0.980718	0.000183	1.010866	0.977028	1.045876	0.533858
ATF3	1.010849	1.007845	1.013861	1.18E‐12	1.002148	0.993916	1.010447	0.610207
CUX2	0.902663	0.866459	0.94038	9.43E‐07	0.990658	0.952535	1.030307	0.639235
ARID5A	1.012701	1.008765	1.016653	2.14E‐10	0.998024	0.989166	1.006962	0.663694
HLF	0.96132	0.944213	0.978737	1.66E‐05	0.991832	0.952778	1.032487	0.689048
TEF	0.951722	0.940059	0.963529	3.67E‐15	1.004908	0.974296	1.036481	0.756434
ZNF217	1.087498	1.063543	1.111993	1.57E‐13	1.005355	0.941372	1.073688	0.873516
ZNF609	0.971	0.948102	0.99445	0.015648	0.996279	0.939781	1.056174	0.900409
NCOA2	0.995695	0.977963	1.013748	0.637908	—	—	—	—
NCOA1	0.989426	0.974981	1.004084	0.156535	—	—	—	—

We further verified the expression profiles of SRF across various GBM datasets, and the findings were largely consistent with those presented in Figures 1B and S1A–C. In the TCGA, Rembrandt, and CGGA301 datasets, the expression levels of SRF in GBM samples were significantly higher than those in LGG samples (Figure 1C). Similarly, SRF expression levels in mesenchymal subtypes from the GSE13041, GSE4412, and GSE53733 datasets were also significantly elevated compared with those in the classical and proneural subtypes (Figure 1D). Furthermore, the study conducted by Johnson et al. identified three novel GBM subtypes based on epigenetic heterogeneity: differentiated‐like, stem‐like, and proliferating stem‐like. Among these, the differentiated‐like subtype exhibited a relatively better prognosis, while the proliferating stem‐like subtype was associated with the highest malignancy and poorest prognosis [16]. We deconvoluted the single‐cell tumor data from Kevin C. Johnson into spatial sequencing data, and through spatial correlation analysis, we found that SRF was predominantly expressed in the proliferating stem‐like subtype (*R* = 0.38), with the lowest expression observed in the differentiated‐like subtype (*R* = −0.19) (Figure 1E). IHC detection of SRF expression in 10 LGG and 80 GBM samples collected by our team further confirmed that SRF was significantly upregulated in GBM than in LGG (Figure 1F). However, SRF did not exhibit significant differences between normal brain tissue and glioma tissue across multiple datasets (TCGA, GSE7696, GSE16011, Rembrandt, and E‐MTAB‐3892), indicating that it may not act a role in oncogenesis (Figure S1D). After classifying glioma samples from the GSE13041, GSE4412, GSE82009, CGGA325, and CGGA693 datasets into SRF high‐expression and low‐expression groups and performing Kaplan–Meier analysis, we discovered that patients with high SRF expression had a significantly shorter OS compared with those with low SRF expression (Figure 1G). CGGA693 cohort was employed to explore the relationship between SRF expression levels and the clinicopathological characteristics including age, gender, histology, WHO grade, IDH mutation, MGMT methylation, 1p19q codeletion, and OS. Of all parameters compared, gender, tumor grade, histology, 1p19q codeletion, and OS were found to significantly correlated with SRF expression (Table S4). Taken together, these solid results demonstrate SRF's critical roles in GBM malignant phenotypes and may serve as a potential target in GBM therapies.

### 2.2 SRF Promotes GBM Progression via Activating Vascular ECs and Enhancing Angiogenesis

**FIGURE 2:**
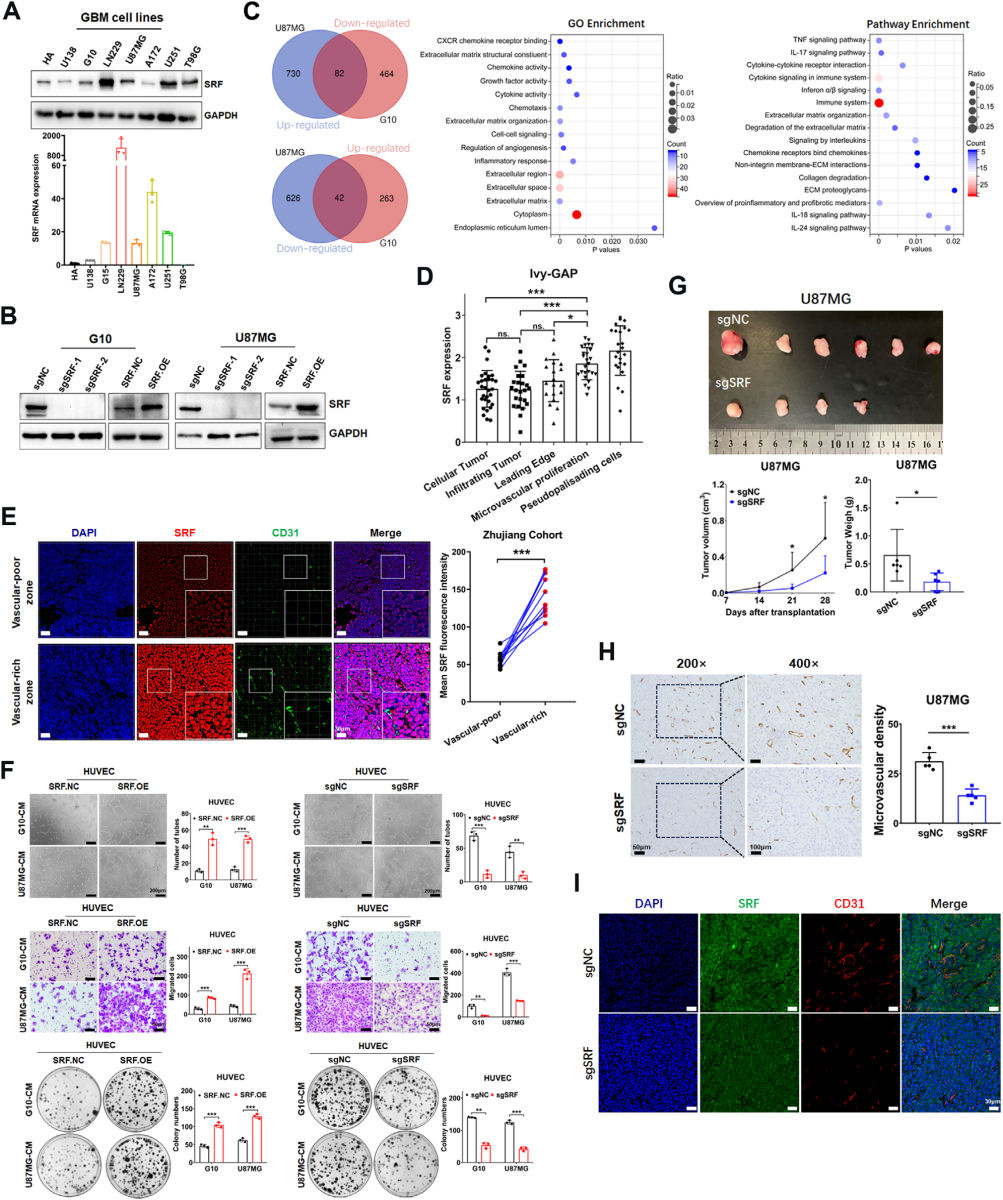
Upregulation of SRF in GBM significantly promotes tumor angiogenesis. (A) Expression of SRF mRNA and protein was detected in HA and seven different GBM cell lines. (B) Western blot assay was used to verify the successful construction of SRF overexpression and knock‐out GBM cells. (C) The Venn diagram illustrates the overlap of DEGs between U87MG and G10 RNA‐Seq data. Pathway and GO enrichment analysis of DEGs were conducted using the overlapping DEGs. (D) Scatter diagram represents SRF expression level in five regions (cellular tumor, infiltrating tumor, leading edge, microvascular proliferation, and pseudopalisading cells) of GBM tissues from the Ivy‐GAP dataset. (E) IF assay analysis compared the difference in SRF expression levels between vascular‐poor and vascular‐rich zones. (F) Tube formation, transwell, and colony formation assays were performed to detect the effect of CM treatment on HUVEC activation. (G) Subcutaneous xenograft tumor model showed the effect of SRF knockout on tumor proliferation. Tumor volume and weight were measured and expressed as mean ± SD. (H) Paraffin‐embedded xenograft sections were stained with antibody targeting human CD31. Microvascular density was compared between the sgNC and sgSRF groups. (I) IF assays showed the SRF expression and vessel density of xenograft sections.

To investigate the function and mechanism of SRF in GBM progression, we first examined the endogenous expression levels of SRF in seven GBM cell lines and normal astrocytes HA via western blot and RT‐qPCR assays (Figure 2A). U87MG and G10 (GBM cell lines constructed by our research groups) cells, which expressed SRF at a moderate level, were chosen to construct stable overexpression and knockout cell lines. The construction efficiency was validated through western blot and RT‐qPCR (Figures 2B and S2D). Subsequently, we performed RNA‐Seq on the aforementioned stable cells and their control cells to analyze transcriptomic changes caused by SRF overexpression/knockout. As shown in Figure S2A,B, 1480 DEGs (812 upregulated and 668 downregulated genes) were identified in the SRF.OE group, 851 DEGs (305 upregulated and 546 downregulated genes) were identified in the sgSRF group. Further GO and KEGG enrichment analyses consistently revealed that these DEGs were significantly enriched in cytokine/chemokine activities, extracellular matrix (ECM), cell adhesion, angiogenesis, immune response, and other related pathways. Enrichment results of DEGs intersection between SRF.OE and sgSRF also reach the same conclusion (Figure 2C). To further confirm the accuracy of the transcriptome sequencing results, we analyzed genes significantly correlated with SRF expression levels in the TCGA‐GBM cohort using the LinkedOmics database. GO and KEGG enrichment analyses of the top 100 correlated genes showed results consistent with RNA‐Seq in cell lines (Figure S2C). The Ivy‐GAP (Ivy Glioblastoma Atlas Project) defines five anatomical features based on histology: the cellular tumor, the infiltrating tumor, the leading edge, the microvascular proliferation zone, and the pseudopalisading cells around necrosis. This project also provides transcriptomic data for each anatomical feature area [17]. We analyzed the expression of SRF in these five regions and found that SRF expressed at the highest level in the microvascular proliferation and pseudopalisading cell regions (Figure 2D). Combining the formation mechanism of tumor vascular microenvironment, these results suggested that SRF might promote GBM malignant progression by mediating angiogenesis. Consistent with our assumption, we observed by IF assay in GBM specimens that samples with high SRF expression displayed higher vessel density relative to those with low SRF expression (Figure 2E). However, the underlying mechanisms remain largely unknown.

The proliferation, migration, and tube formation of ECs are crucial steps in tumor angiogenesis. Based on this, we first evaluated the effects of SRF on the proliferation and migration abilities of human umbilical vein ECs (HUVECs) in vitro. To intuitively detect the effect of SRF on GBM angiogenesis, culture media (CM) from SRF overexpression/knockout cells and their control groups were collected to treat HUVEC cells for 24 h, followed by tube formation, transwell, and colony formation assays (Figure 2F). The results showed that HUVEC cells treated with CM from SRF overexpression U87MG and G10 cells had dramatically more tube formation, transwell chamber cells migration, and colony formation than the control group; the results were opposite for SRF knockout. Furthermore, we conducted a subcutaneous tumor model to explore the effect of SRF overexpression/knockout on the growth of U87MG cells in vivo. The results showed that SRF overexpression significantly promoted the growth of U87MG subcutaneous tumors (Figure S2E), while SRF knockout exhibited the opposite results (Figure 2G). IHC (Figures S2F and 2H) and IF assays (Figures S2G and 2I) both revealed that the tumor microvascular density displayed a positive correlation with SRF expression. These results collectively indicate that SRF promotes GBM angiogenesis via activating vascular ECs, whereas the specific molecular mechanism remains to be further elucidated.

### 2.3 SRF Promotes Angiogenesis by Directly Upregulating OLFML3 and Remodeling ECM

**FIGURE 3:**
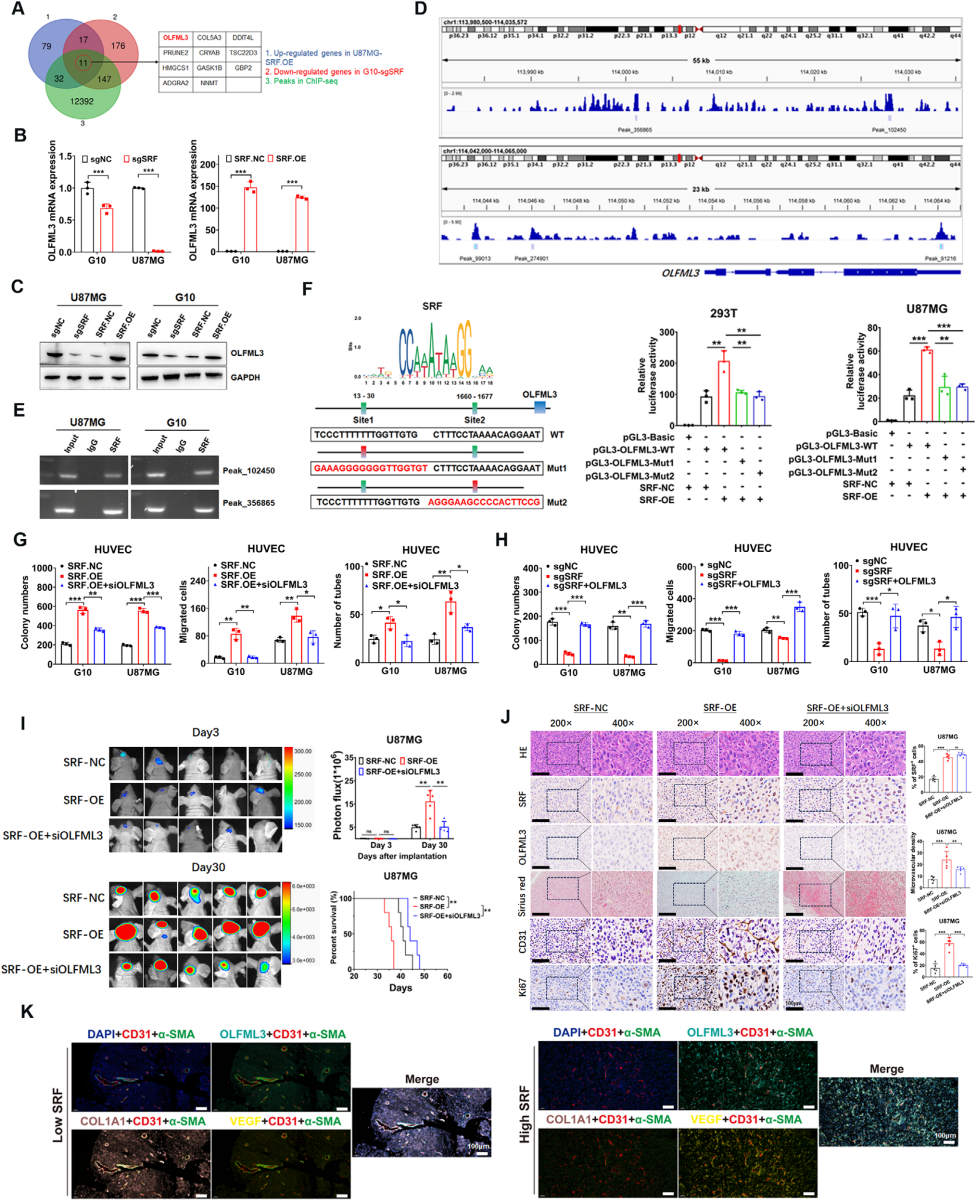
SRF promotes angiogenesis by regulating OLFML3 transcriptional upregulation. (A) The Venn diagram indicated 11 overlapped genes in RNA‐Seq and ChIP‐Seq of SRF. (B) An RT‐qPCR assay was conducted to verify the effect of SRF on OLFML3 transcription in U87MG and G10 cells. (C) A Western blot assay was conducted to verify the effect of SRF on OLFML3 protein expression. (D) Representative ChIP peak located on SRF, which is directly targeted on gene OLFML3. (E) Amplification of SRF‐binding sites peak_102450 and peak_356865 after ChIP assay was indicated in the agarose gel. (F) Potential binding sites of SRF in OLFML3 promotor were predicted by JASPAR 2020. OLFML3 promotor‐luciferase wild‐type or mutated reporters were cotransfected with PGL3–SRF, and then luciferase reporter assays were performed. (G and H) Effects of OLFML3 siRNA (G) and expression vector (H) on SRF‐mediated HUVEC activation through colony formation, transwell, and tube formation assays. Data were presented as mean ± SD. (I) Effects of OLFML3 siRNA on SRF‐mediated tumor progression through an orthotopic xenograft model. Representative bioluminescent images of xenografts at Days 3 and 30 after tumor cell implantation were shown in the left panel. The fluorescence signal intensity of xenografts and K–M survival analysis of mice bearing xenografts were shown in the right panel. (J) Paraffin‐embedded xenograft sections were stained with Sirius Red or antibodies targeting human SRF, OLFML3, Ki67, and CD31. (K) IF staining of OLFML3, CD31, α‐SMA, COL1A1, and VEGF in GBM tissues with high and low SRF expression. Scale bar represents 100 µm.

Considering the functional characteristics of SRF as a TF, we performed ChIP‐Seq to analyze the DNA sequences bound by SRF. Intersected with the DEGs from RNA‐Seq (Figure S3A), we identified 11 genes directly upregulated (Figure 3A) and 18 genes downregulated by SRF (Figure S3B). Among the 29 overlapping genes, olfactomedin‐like 3 (OLFML3) is most closely related to ECM, cell adhesion, angiogenesis, and other microenvironmental characteristics. RT‐qPCR (Figure 3B) and western blot (Figure 3C) assays further confirmed that SRF overexpression upregulates OLFML3 expression, while SRF knockout has the opposite effect. The correlation between SRF and OLFML3 expression in GBM specimens was analyzed in multiple datasets, including CGGA301, CGGA325, CGGA693, and TCGA (Figure S3C). The results revealed that OLFML3 was positively correlated with SRF expression. These results collectively suggest that OLFML3 serves as the key downstream gene for SRF's function.

As shown in Figure 3D, we noticed that unlike the traditional model where TFs bind to the promoter region, the signal peaks of SRF on the OLFML3 gene are mainly located in the intron and distal intergenic regions, with six binding peaks in total, five of which are located on the transcript ENST00000633022.1 (Table S5). We selected two intron regions, Peak_356865 and Peak_102450, for further validation, and DNA gel electrophoresis confirmed the binding of SRF to these two regions (Figure 3E). Although no SRF binding peaks were detected in the promoter region of OLFML3, we still explored the possibility through dual‐luciferase reporter assay. Using the JASPAR database, we predicted the SRF binding sites in the OLFML3 promoter region and identified multiple high‐confidence sites (Table S6), with the highest two located at 13–30 bp (site 1) and 1660–1677 bp (site 2). Subsequently, we constructed a dual‐luciferase reporter gene vectors with these two sites mutated and wild‐type vectors, as shown in the schematic diagram in Figure 3F. The dual‐luciferase reporter assay demonstrated that SRF could bind to both site1 and site2 in 293T and U87MG cells (Figure 3F).

Studies have shown that OLFML3 is a secreted ECM glycoprotein with a C‐terminal olfactomedin domain that promotes protein–protein interactions, cell adhesion, and intercellular interactions. It functions as both a scaffold protein and a proangiogenic vascular tissue remodeling protein. We analyzed the expression characteristics and clinical significance of OLFML3 in CGGA and TCGA datasets, and the results consistently showed that OLFML3 expression significantly increases with tumor grade (Figure S3D). In Ivy‐GAP data, OLFML3 expression is highest in the microvascular proliferation region, significantly higher than in other regions (Figure S3E). Furthermore, K–M analysis of multiple large sample datasets in CGGA and TCGA showed that high OLFML3 expression is significantly associated with poor prognosis in patients (Figure S3F). These results suggest OLFML3's significant roles in malignant phenotypes (especially angiogenesis) and poor prognosis of gliomas. To validate the roles of OLFML3 in SRF's proangiogenic function, we constructed its overexpression vectors and siRNA for subsequent rescue experiments. The overexpression/knockdown efficiency in GBM cell lines U87MG and G10 was validated via western blot (Figure S3G). In vitro and in vivo rescue experiments were then conducted. As shown in Figures 3G and S3H, OLFML3 knockdown markedly inhibited the ECs activation (proliferation, migration, and tube formation abilities) mediated by SRF overexpression. Conversely, restoring OLFML3 expression significantly restored the ECs inactivation caused by SRF deficiency (Figures 3H and S3I). The intracranial xenograft model further revealed that knockdown of OLFML3 in U87MG cells dramatically inhibited the accelerated tumor progression and reduced survival in mice caused by SRF overexpression (Figure 3I). Histological staining of xenograft sections indicated that Ki67‐positive cells, CD31 density, and Sirius Red staining intensity (indicator of collagen fibers in ECM) were also significantly inhibited after OLFML3 knockdown (Figure 3J). To further validate that OLFML3 activates ECs by degrading/remodeling the ECM, we conducted multiplex immunofluorescence staining (OLFML3, CD31 [ECs marker], α‐SMA [pericytes marker], COL1A1 [major component of ECM] and VEGF) using GBM specimens. As shown in Figure 3K, compared with the low SRF group, GBM tissues with high SRF expression exhibit higher OLFML3 content, accompanied with higher CD31, α‐SMA and VEGF level and lower COL1A1 (major component of ECM) content. To clarify whether OLFML3 is expressed by ECs or if it is solely secreted by tumor cells, we first conducted western blot analysis to compare the protein level of OLFML3 in HUVEC and GBM cell lines, and the results revealed that OLFML3 expression is the lowest in HUVECs (Figure S3J). Consistently, single‐cell sequencing of GBM samples from GSE194329 dataset demonstrated markedly reduced OLFML3 expression in ECs, B cells, and T cells relative to other cell types (Figure S3K). These findings collectively suggest that OLFML3 is predominantly secreted by tumor cells. Further investigation into the impact of OLFML3 knockdown specifically in ECs—assessed through transwell migration and tube formation assays—showed no significant effect on their activation status (Figure S3L). These results fully demonstrate that OLFML3 plays a key role in SRF‐mediated angiogenesis and tumor progression.

### 2.4 P54nrb May Serve as the Key Cofactor by Directly Interacting With SRF

**FIGURE 4:**
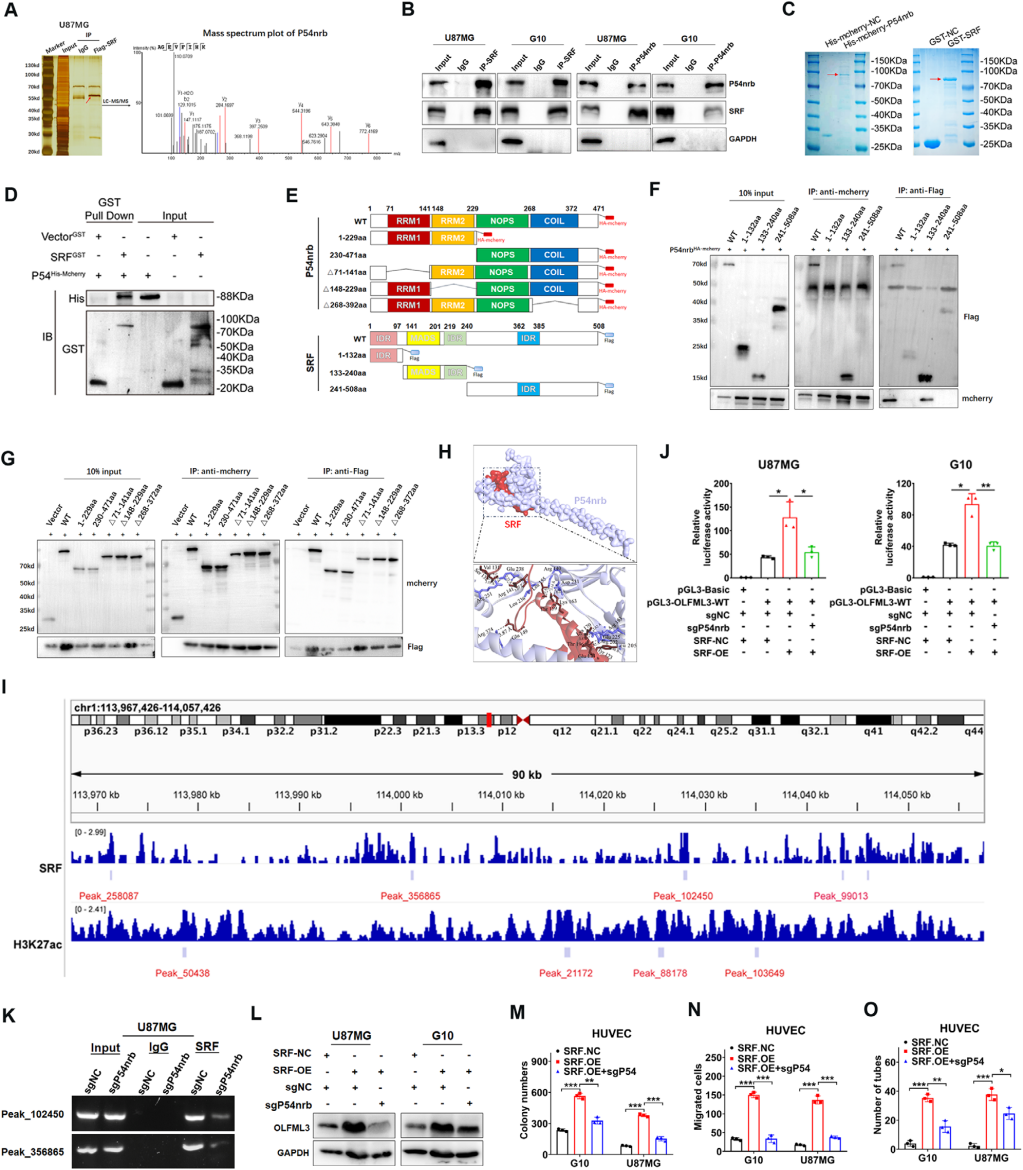
P54nrb directly interacts with SRF and may serve as its transcription cofactor. (A) Immunoprecipitation and silver staining were performed by using U87MG–SRF–OE cell lysate with the anti‐Flag antibody. The spectrogram of the differential protein band was identified as P54nrb (quadrupole mass spectrometer). (B) Co‐IP analysis of the interactions between SRF and P54nrb in U87MG and G10 cells. (C and D) Direct binding of recombinant protein GST–SRF to His–mcherry–P54nrb using GST pull‐down assay. (E) Diagrammatic representation of SRF (below panel) and P54nrb (above panel) proteins and their truncated forms. (F and G) 293T cells were transfected with the indicated truncation constructs and subjected to immunoprecipitation with anti‐Flag (against SRF) and mcherry (against P54nrb). (H) Molecular docking analysis of the SRF–P54nrb complex. Residues involved in the interaction interface are shown as sticks. SRF and P54nrb are colored in silver and red, respectively. (I) ChIP‐seq data of the U87MG cell line showing SRF and H3K27ac peaks enriched at the genome of OLFML3. (J) Dual luciferase reporter assays were performed to detect the effect of P54nrb knockout on the SRF mediated OLFML3 upregulation in U87MG and G10 cells. (K) A ChIP assay was conducted to detect the effect of P54nrb knockout on the interaction between SRF and OLFML3 genome in U87MG cells. (L) Western blot was conducted to detect the effect of P54nrb knockout on SRF‐mediated OLFML3 upregulation in G10 and U87MG cells. (M–O) Effects of P54nrb knockout on SRF‐mediated HUVEC activation through colony formation assay (M), transwell assay (N) and tube formation assay (O). Data were presented as mean ± SD.

To further investigate the concrete mechanism by which SRF upregulates OLFML3 expression, we sought to identify the proteins that interacted with SRF. Flag‐tagged SRF was purified from U87MG cells, and the interacted proteins were identified by silver staining. A significant differential band between 55 and 70 kDa was observed and subjected to mass spectrometry (LC/MS–MS) analysis, identifying P54nrb as the key interacting protein of SRF (Figure 4A). To verify their interaction, we performed Co‐IP in U87MG and G10 cells using antibodies against SRF and P54nrb. Western blot analysis confirmed the presence of both SRF and P54nrb in the precipitate products (Figure 4B). IF staining of SRF and P54nrb in U87MG and G10 cells both showed significant colocalization under confocal laser scanning microscopy (Figure S4A). We then purified the GST‐SRF protein and His–mcherry–P54nrb protein in vitro (Figure 4C) and confirmed their specific direct binding by GST‐Pulldown experiments (Figure 4D). Next, we investigated the protein domains mediating the interaction between SRF and P54nrb by generating Flag‐tagged and HA–mcherry‐tagged truncation mutants based on the protein structure, respectively (Figure 4E). As shown in Figure 4F,G, the results revealed that the 133–240aa domain of SRF is essential for the binding of SRF with P54nrb. Further protein–protein docking models also reach a consistent conclusion (Figure 4H).

Given that P54nrb generally function as a transcription cofactor that may gather near the enhancer region to promote TF interaction with promoters, thus markedly activating gene expression, H3K27ac ChIP‐seq was conducted using U87MG cells, and the peaks on OLFML3 were compared with the peaks of SRF. The results revealed that Peak_102450 of SRF is very close to peak_88178 and peak_103649 of H3K27ac on the OLFML3 gene, confirming the relationship between the SRF/P54nrb complex and OLFML3 enhancers (Figure 4I and Table S7). We further investigated the role of P54nrb in SRF‐mediated angiogenesis through rescue experiments. First, we constructed P54nrb sgRNA and transfected it into U87MG and G10 cells. Western blot analysis confirmed the successful knockout of P54nrb expression (Figure S4B). Next, P54nrb–sgRNA was transfected into the SRF–OE groups of U87MG and G10 cells. Dual‐luciferase reporter (Figure 4J) and ChIP (Figure 4K) assay were conducted and consistently showed that SRF at the OLFML3 genome was significantly reduced when P54nrb was depleted by sgRNA. The expression changes of OLFML3 were assessed using western blot and the results demonstrated that P54nrb knockout significantly reversed the SRF‐mediated upregulation of OLFML3 (Figure 4L). Consistent findings were obtained from subsequent colony formation (Figures 4M and S4C), transwell (Figures 4N and S4D), and tube formation (Figures 4O and S4E) assays that knocking out P54nrb markedly reversed the SRF‐mediated activation of ECs.

### 2.5 P54nrb Acts Critical Roles in SRF Mediated Angiogenesis and Promotes SRF Phase Separation in the Nucleus

**FIGURE 5:**
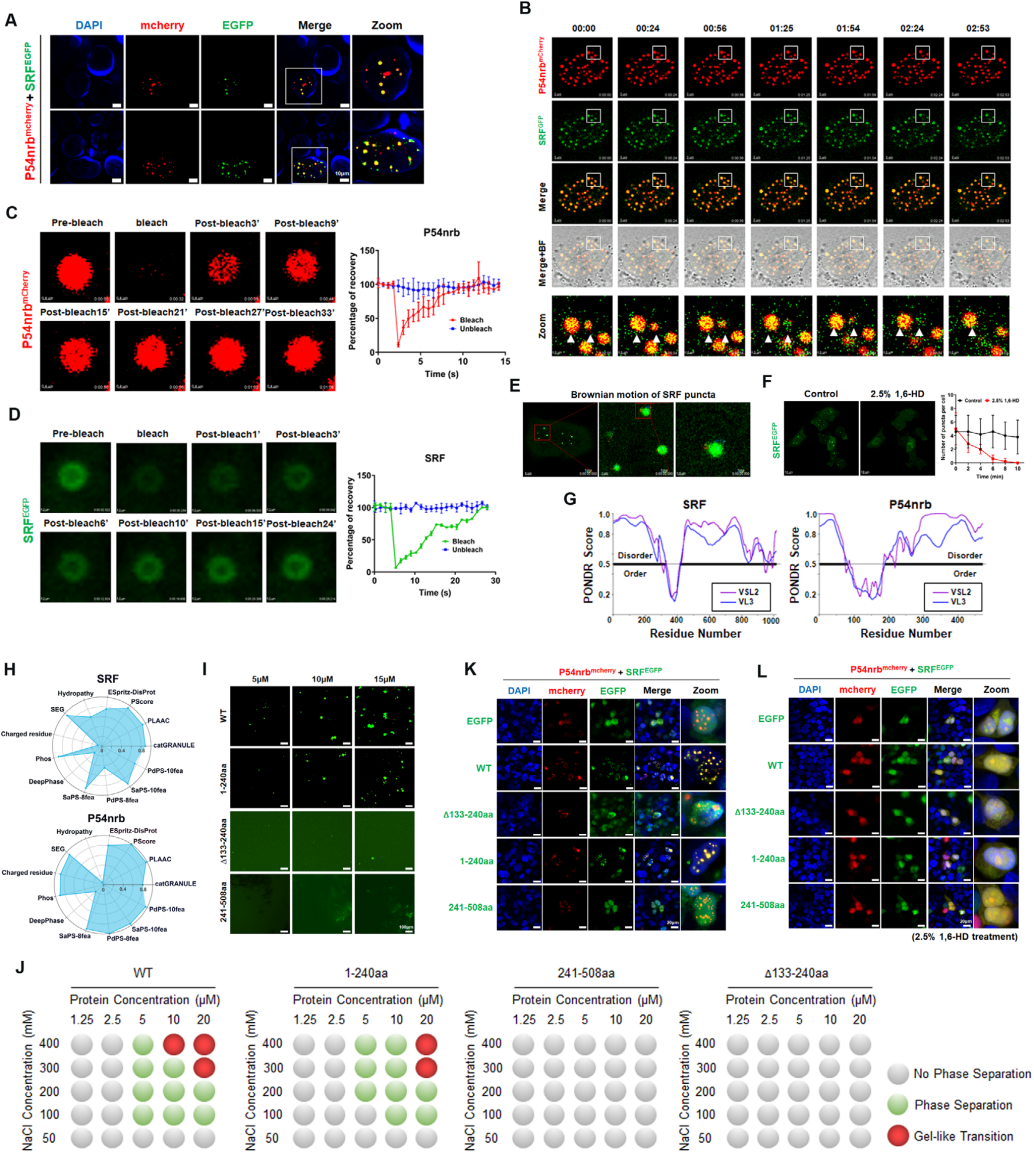
P54nrb acts critical roles in SRF mediated angiogenesis and promotes SRF phase separation in the nucleus. (A) Confocal microscopy images showing the colocalization of SRFGFP and P54nrbmcherry in the nucleus of 293T cells. (B) Time‐lapse imaging of nuclear puncta fusion of mCherry–P54nrb and GFP–SRF. (C and D) FRAP recovery experiment of mCherry–P54nrb (C) and EGFP–SRF (D) puncta. The right panel represents quantification analysis of the FRAP recovery experiment. (E) Recording of the track of the motion of EGFP–SRF puncta over the course of 100 min. Scale bar, 2 µm. (F) Live cell images showing effect of 2.5% 1,6‐hexanediol (1,6‐HD) incubation on SRF puncta formation. Right panel represents quantities of EGFP–SRF puncta versus time for untreated control and 1,6‐HD incubation. Scale bar, 2 µm. (G) The IDRs of SRF and P54nrb were detected by the Predictor of Natural Disordered Regions (PONDR) database. (H) Prediction of phase separation ability of SRF and P54NRB proteins by PhaSepDB. (I) Images of SRF–WT/∆133–240aa/1–240aa/241–508aa droplets formed in vitro. Scale bar, 5 µm. (J) Phase diagrams of SRF–WT/∆133–240aa/1–240aa/241–508aa proteins (ranging from 1.25 to 20 µM) incubated in 50 mM NaH_2_PO_4_ (pH 7.5), 10% (w/v) PEG 8000, and sodium chloride (ranging from 50 to 400 mM). Gray dots, no phase separation; green dots, phase separation; red dots, gel‐like transition. (K and L) Confocal microscopy images showing the colocalization of P54nrb and SRF truncations with (L) or without (K) 1,6‐HD treatment.

Using fluorescence confocal microscopy, we unexpectedly discovered that SRF and P54nrb distributed as scattered nucleus puncta in certain regions of U87MG and G10 cells (Figure S4A). Cotransfection of EGFP–SRF and mCherry–P54nrb into 293T cells revealed a more pronounced phenomenon, with some cells showing distinct droplet‐like aggregation (Figure 5A). Continuous live‐cell dynamic imaging for approximately 3 min showed that these droplets exhibited the key features of phase‐separated condensates, including high sphericity and fusion behavior (Figure 5B). Along with the observable fusion, these puncta increased in size over time (Video S1). Further fluorescence recovery after photobleaching (FRAP) assays further revealed that the P54nrb (Figure 5C and Video S2) and SRF (Figure 5D and Video S3) puncta formed partially recovered after photobleaching in live cells. Motion type analysis revealed that the SRF puncta constantly underwent confined Brownian motion (Figure 5E and Video S4). We also examined the sensitivity of SRF puncta to 1,6‐hexanediol (1,6‐HD), a compound recognized to disrupt LLPS droplets, and found that 1,6‐HD caused a significant SRF puncta reduction (Figure 5F). The presence of intrinsically disordered regions (IDRs) is the structural basis for phase separation. We analyzed the amino acid sequences of SRF and P54nrb using PONDR. The results showed that both proteins have a substantial presence of IDRs in their structures. Besides, SRF was ordered only in the region of approximately 150–210aa, which coincides with the key domain for its interaction with P54nrb (Figure 5G). The phase separation potential of the two proteins was further examined by PhaSePred, a prediction tool based on multiple essential characteristics of phase separation, including IDRs, low complexity domains, hydropathy, coiled‐coil structure, and so on [18]. As shown in Figure 5H and Table S8, SRF and P54nrb both exhibited high potential to trigger phase separation independently or passively. These results collectively substantiated the phase separation potential of these two proteins, whereas the downstream effects require further exploration.

To further unveil the critical domain of SRF driving LLPS, we purified recombinant SRF–WT/∆133–240aa/1–240aa/241–508aa proteins in vitro. Liquid‐like‐droplet‐formation assay revealed that only SRF–WT and SRF–1–240aa but not another two isoforms could form droplets in the presence of 10% PEG‐8000 (Figure 5I). Phase diagrams were generated based on the results of the droplet formation assays (Figure 5J), showing enhanced LLPS of both SRF–WT and SRF–1–240aa with increasing protein concentration and sodium chloride concentration (within physiological levels), indicating that electrostatic interactions contributed to SRF droplets formation. To further validate the key domain of SRF interacting with P54nrb to form puncta, mcherry‐tagged P54nrb–WT and EGFP‐tagged SRF–WT or SRF truncations were cotransfected in 293T cells. Fluorescence confocal microscope observation indicated that only SRF–WT and SRF–1–240aa are capable of forming puncta with P54nrb, consistent with the droplet formation assays (Figure 5K). Importantly, droplets formed by SRF–WT/1–240aa and P54nrb in vivo could also be disrupted by 1,6‐HD (Figure 5L). These results together suggested that SRF–133–240aa is the essential domain for the SRF/P54nrb phase separation. Taken together, the results above comprehensively elucidated the concrete mechanisms by which SRF/P54nrb form transcription complexes and activate angiogenesis in GBM.

### 2.6 SRF/P54nrb Complex‐Mediated Vascular Hyperplasia Inhibits Immune Infiltration

**FIGURE 6:**
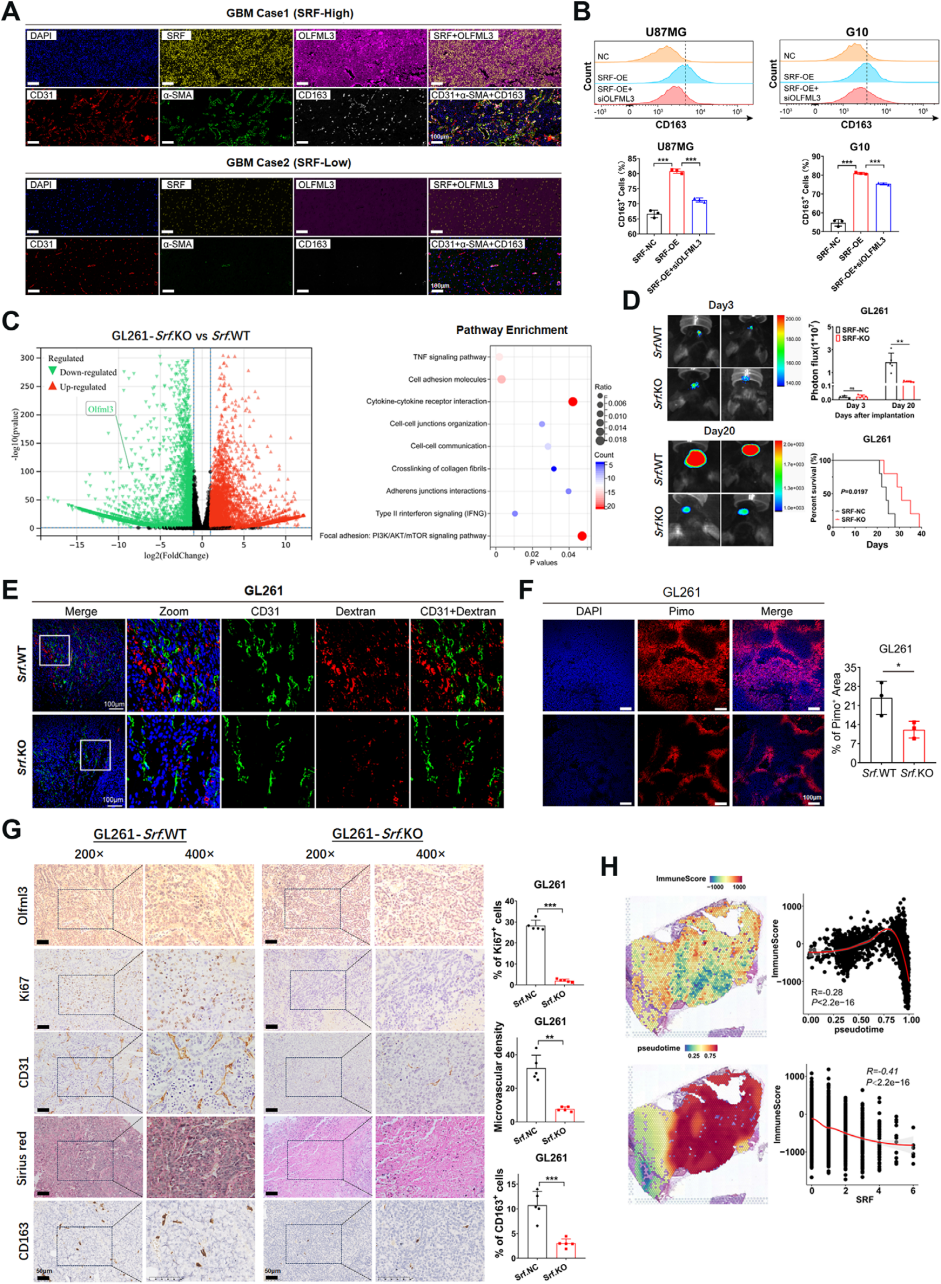
SRF/P54nrb complex‐mediated vascular hyperplasia inhibits immune infiltration. (A) IF staining of SRF, OLFML3, CD31, α‐SMA, and CD163 in GBM tissues with high and low SRF expression. Scale bar represents 100 µm. (B) THP‐1 were cocultured with indicated cells, and FACS analyses show the effect of SRF upregulation and OLFML3 knockdown on the percentage of CD163^+^ cells. (C) The volcano plot represents all DEGs between the Srf.KO and Srf.WT GL261 cells. The red dot represents upregulated genes, and the green dot represents downregulated genes. Pathway enrichment analysis was conducted using the DEGs. (D) Effects of Srf knockout on tumor progression through an orthotopic xenograft model. Representative bioluminescent images of xenografts at Days 3 and 20 after tumor cell implantation were shown in the left panel. The fluorescence signal intensity of xenografts and K–M survival analysis of mice bearing xenografts were shown in the right panel. (E) Tumor‐vasculature leakiness of Srf.KO and Srf.WT groups was measured by dextran. (F) Tumor hypoxia of Srf.KO and Srf.WT groups was measured by pimonidazole. (G) Paraffin‐embedded xenograft sections were stained with Sirius Red or antibody targeting mouse OLFML3, CD31, Ki67, and CD163. (H) Correlation between SRF expression and immune score in GBM spatial transcriptome data (GSE194329).

To further investigate whether SRF‐mediated angiogenesis affects the immune microenvironment in GBM, we collected 80 clinical GBM samples and performed IHC staining of SRF to divide the samples into high/low SRF expression groups. Next, we conducted multiplex immunofluorescence staining (SRF, OLFML3, CD31, α‐SMA, and CD163 (M2‐polarized macrophage marker)) to detect the distribution of blood vessels and M2‐polarized macrophages in GBM tissues with different SRF expression levels (Figure 6A). The results showed that compared with the low SRF group, GBM tissues with high SRF expression presented significantly higher levels of OLFML3 expression, and the quantities of microvascular and M2‐polarized macrophage were markedly increased, then was accompanied by a decrease in perivascular cell coverage (α‐SMA/CD31), indicating excessive angiogenesis, vascular structural abnormalities and infiltration of M2‐polarized macrophages. Moreover, in line with the mainstream opinion [19], CD163^+^ cells were mainly distributed around the vascular regions, indirectly reflecting the close relationship between vasculature and the immune microenvironment in GBM. Further flow cytometry analysis using macrophage‐like THP‐1 cells confirmed that SRF dramatically increased the number of CD163^+^ cells, which could be reversed by knocking down OLFML3 (Figure 6B).

To explore the impact of SRF on the immune microenvironment, we first successfully constructed an *Srf* knockout cell line using the GL261 cell line. RNA‐seq revealed that, consistent with the results from human GBM cell lines U87MG and G10, *Srf* knockout also led to a downregulation of *Olfml3* expression. GO and pathway enrichment analyses also showed that the DEGs were significantly enriched in cytokine/chemokine activity, ECM, angiogenesis, and so on (Figure 6C). The GSEA results reflected consistent results (Figure S5). Subsequently, we performed intracranial orthotopic transplantation utilizing the aforementioned GL261 cells in C57 mice. Tumor size was monitored using bioluminescence imaging, and OS was recorded. The results showed that *Srf* knockout dramatically inhibited tumor growth and prolonged survival time of the tumor‐bearing mice (Figure 6D). Excessive angiogenesis in GBM is usually accompanied by malformed vascular structures and hypofunction, leading to vascular leakage and the release of large amounts of substances (including cytokines, chemokines, etc.) into the microenvironment, promoting the infiltration of immune‐suppressive cells such as M2‐polarized macrophages (Consistent with the multiple immunofluorescence results in Figure 6A). Therefore, the fluorescent tracer PI‐dextran was employed to detect the function of *Srf* on GBM vascular permeability. The results showed that both vascular density and dextran leakage were significantly lower in the Srf.KO group compared with the Srf.WT group (Figure 6E), indicating *Srf* knockout could effectively impede the vascular leakiness. Furthermore, *Srf* knockout also reduced tumor hypoxia, as demonstrated by the substantial decrease in the pimonidazole (Pimo)‐labeled hypoxic areas (Figure 6F). IHC staining of xenograft tissues showed that the olfml3 expression, vessel densities, and positive rates of Ki67 and CD163 were significantly lower in the *Srf*.KO group. Sirius Red staining also showed that collagen fibers of the Srf.KO group were much denser (Figure 6G). After completing the animal experiments, we further analyzed the relationship between SRF and immune infiltration in GBM specimens using bioinformatics methods. Estimate and SpaceFlow R packages were used to analyze immune score and pseudotime on spatial sequencing data. Subsequent correlation analysis showed that the trend of SRF changes was opposite to the immune score, with a correlation coefficient of −0.41 (Figure 6H). These results collectively indicate that Srf knockout significantly reduces ECM degradation and inhibits angiogenesis and the infiltration of M2‐polarized macrophages in tumor tissues, thereby inhibiting the rapid progression of GBM.

### 2.7 Correlation Between SRF Expression and Vascular Microenvironment Parameters in GBM Specimens

**FIGURE 7:**
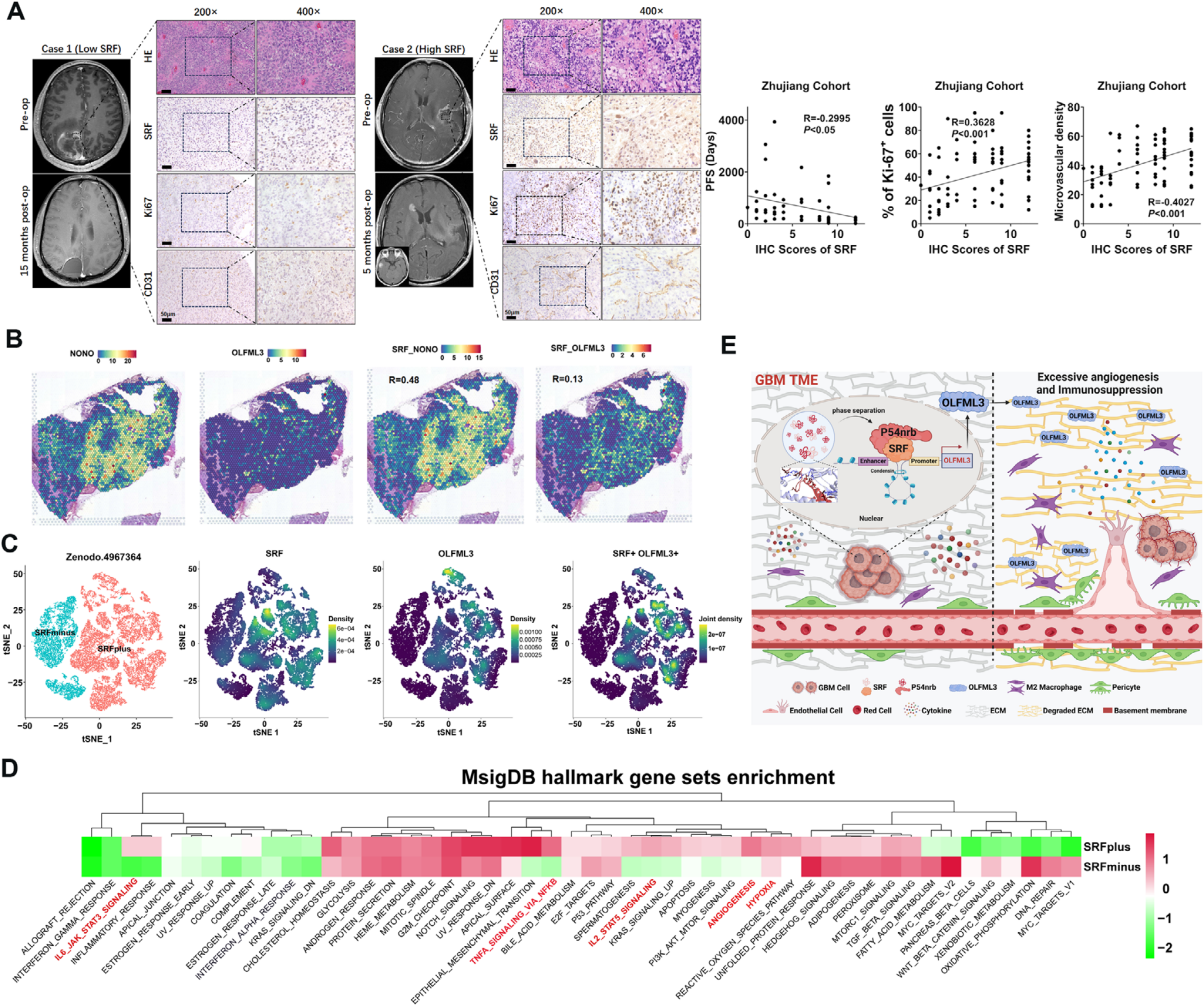
Correlation between SRF expression and vascular microenvironment parameters in GBM specimens. (A) Representative magnetic resonance imaging (MRI) of low SRF expression and high SRF expression GBM tumors before and after standard treatment. HE, SRF, Ki67, and CD31 staining results were also shown. Op, operation. Correlations between SRF expression and PFS, Ki67 positive rate, and microvascular density were presented. PFS, progression‐free survival. (B) Coexpression of SRF and NONO, OLFML3 were analyzed in GBM spatial transcriptome data (GSE194329). (C) Tumor cells in Zenodo.496364 were categorized into SRFplus and SRFminus groups based on the median SRF expression. Joint density plots indicated the significant coexpression of SRF and OLFML3. (D) DEGs between SRFplus and SRFminus groups were employed in GSEA analysis using the MsigDB hallmark gene sets. (E) Mechanism model of the SRF/P54nrb/OLFML3 axis in the formation of GBM vasculature microenvironment.

Finally, we performed bioinformatics and IHC analyses to determine whether the highly vascularized and immunosuppressive microenvironment regulated by the SRF/P54nrb/OLFML3 axis in our cell models was also credible in GBM specimens. As shown in Figure 7A, GBM specimens with high SRF expression levels showed faster imaging progression. IHC scores of SRF were negatively correlated with PFS (*R* = −0.2995, *p* < 0.05), and positively correlated with Ki67 positive rate (*R* = 0.3628, *p* < 0.001) and vascular density (*R* = 0.4027, *p* < 0.001). Positive correlation between SRF and OLFML3, NONO (the encoding gene of P54nrb), was also validated in spatial transcriptome data (Figure 7B). From the GBM single‐cell RNA (scRNA) data available in Zenodo.496364, we extracted tumor cell data and categorized them into two groups based on the median SRF expression level: the minus group (below median) and the plus group (above median). Consistent with our findings, OLFML3 expression was significantly lower in the SRFminus group than the SRFplus group (Figure S6A). The joint density plot further revealed a dramatic coexpression between SRF and OLFML3 (Figure 7C). We then conducted DEG analysis between the SRFminus and SRFplus groups. Hallmark gene set enrichment also revealed significant enrichment of multiple angiogenesis‐related pathways such as ANGIOGENESIS and HYPOXIA (Figure 7D). Intercellular communication networks between GBM cells and other cellular components in the microenvironment were analyzed leveraging the LIANA R package in the two groups. The results showed that in the two groups of cells with different SRF expression levels, the differences in cell communication in the microenvironment also exhibited significant disparities (Figure S6B–D). Moreover, we also screened for the potential TFs upregulating SRF using single‐cell regulatory network inference and clustering (SCENIC) analysis of hGBM scRNA sequencing (scRNA‐seq) data from Zenodo.496364 (Figure S7A). MAX, THRA, and HDAC2 were identified with the lowest regulon activity score for the SRFplus group, while ETV6, IRF2, and JUND were identified with the highest regulon activity (Figure S7B). Further experiments were required to determine which TFs accounted for the overexpression of SRF in GBM. These data further confirm the vital role of the SRF/P54nrb/OLFML3 axis in shaping the vasculature microenvironment and promoting malignant progression of GBM.

## 3 Discussion

GBM (IDH wild type, WHO grade IV) has a postoperative recurrence rate exceeding 90%, and there is currently a lack of effective clinical treatment options. Increasing research indicates that GBM exhibits a high degree of intratumoral heterogeneity, reflected in various aspects such as epigenetics, transcriptomics, metabolomics, proteomics, and the tumor microenvironment. This heterogeneity leads to the coexistence of different GBM phenotypic states, which interact with and depend on the continuously evolving tumor microenvironment, constituting key reasons for GBM's therapeutic resistance and malignant progression [20]. Therefore, identifying more efficient and specific therapeutic targets based on heterogeneity is a significant and challenging focus in current GBM research, and it remains a long‐term endeavor. TFs are terminal effector molecules of aberrant signaling networks in tumor cells, and their dysregulated expression and function are implicated throughout tumorigenesis and progression. Increasingly, it is believed that targeting TFs may be a more specific and efficient therapeutic strategy, potentially minimizing impacts on normal cellular physiological functions and reducing treatment‐related side effects [21]. In this study, by analyzing gene expression profiles and differences of GBM/LGG and mesenchymal/proneural groups from multiple large‐sample datasets, we identified SRF as a key TF mediating GBM malignant phenotypes and poor prognosis. Subsequent in vitro and in vivo experiments proved that SRF promoted angiogenesis and immunosuppression via complex interaction with cofactor P54nrb to upregulate OLFML3.

Verhaak et al. conducted a consensus clustering analysis of transcriptome and genome data from TCGA–GBM, classifying GBM into four subtypes for the first time: mesenchymal, classical, proneural, and neural. Each subtype exhibits specific gene expression patterns [22]. Subsequent studies excluded the interference of normal neurons, thus eliminating the neural subtype. Among the three subtypes, the mesenchymal subtype has the highest malignancy and the worst prognosis, while the proneural subtype has a relatively better prognosis. This research significantly deepened the academic understanding of GBM heterogeneity and laid the foundation for extensive and in‐depth studies. In this study, we examined the gene expression profiles differences among gliomas with varying malignancy degrees to identify key TFs involved in the malignant phenotype of GBM. Analysis of multiple bulk expression profile datasets revealed that SRF is not only significantly overexpressed in GBM compared with LGG but also predominantly expressed in the mesenchymal subtype, the most malignant subtype of GBM. Among the 12 candidate TFs screened, SRF was the only one validated as an independent prognostic factor through two large‐sample datasets. While some studies have preliminary reported the roles of SRF in cancers such as gastric and liver cancer, no direct reports have addressed its role in GBM. Our findings elucidate for the first time the critical role of SRF in the malignant phenotype and poor prognosis of GBM, underscoring its research values.

French et al. performed RNA‐seq on paired primary‐recurrent GBM resections from patients undergoing standard treatment and found that the three abovementioned subtypes formed an interconnected continuous structure in a two‐dimensional space. These subtypes can transform into each other during GBM progression and recurrence, preferentially evolving toward the mesenchymal subtype, which has the highest malignancy and poorest prognosis. This transformation is primarily achieved through microenvironmental remodeling rather than molecular evolution of tumor cells [15]. This underscores the critical roles of the tumor microenvironment in GBM progression. Vascular hyperplasia is a typical feature of GBM, and vascular microenvironment is a crucial component of GBM microenvironment. The ECM acts critical roles in GBM angiogenesis, during which ECM undergoes degradation and remodeling through the action of proteases such as matrix metalloproteinases (MMPs), facilitating EC migration and the formation of lumen‐like structures. Simultaneously, various cytokines stored in the ECM are released, bind to receptors on ECs, activate these cells, and promote their proliferation, migration, and lumen‐like structure formation [23]. Studies have demonstrated that during embryonic development, OLFML3 plays a crucial role in cell adhesion, migration, and differentiation by promoting the binding of BMP‐1/Tolloid proteases and chordin in the ECM, thereby enhancing chordin degradation [24]. BMP‐1, a member of the Tolloid protease family within the MMP superfamily, possesses hydrolytic activity and can cleave or degrade various protein substrates, thereby regulating ECM degradation and remodeling. However, there are currently no reports on the involvement of OLFML3 in ECM degradation and remodeling in tumor tissues. Herein, we found that collagen fibers in tumor tissues with SRF overexpression are sparser, suggesting that OLFML3‐mediated ECM degradation may be a key mechanism by which SRF promotes angiogenesis.

Enhancers are small regions on DNA that can bind with TFs. They influence gene transcription by binding to specific TFs or other transcription elements, precisely controlling the spatiotemporal expression of genes. Enhancers can be situated upstream, downstream, or within intronic regions of their target genes and do not need to be near the transcription start site to regulate gene transcription. Some enhancers are located hundreds of thousands of bases away [25, 26 ]. The structure of the eukaryotic chromatin complex can exist in a supercoiled form, so that even if the enhancers and their target genes are separated by long nucleotide sequences, they remain spatially proximate, thereby increasing the likelihood of enhancers binding to TFs and RNA Polymerase II. Notably, specific TFs can be highly concentrated in the enhancer regions through a phase separation process, promoting their interaction with promoters to form transcriptional activation complexes, thereby greatly promoting gene expression [27, 28 ]. In this study, we found that the binding sites of SRF on the OLFML3 genome included not only the promoter region but also multiple sites in the intronic and distal intergenic regions. H3K27ac ChIP‐Seq analysis indicated that these sites were located very close to several enhancers. Therefore, we hypothesize that this process may involve enhancer regulatory mechanisms.

Additionally, we identified P54nrb as a key cofactor of SRF and elucidated their interaction details at the domain level. This study also first proposed that the SRF/P54nrb complex is assembled via LLPS: Protein structure analysis and observation of subcellular localization indicated that the SRF/P54nrb complex might form phase separation condensates, which was further verified via in vitro and cellular assays. Knocking out P54nrb markedly weakened the transcription activity and proangiogenic function of SRF, which emphasized its crucial roles in regulating phase separation. Functional domains such as IDRs are indispensable for LLPS via mediating oligomerization and/or facilitate the multivalent interactions. Herein, 133–240aa of SRF was identified as the molecular switch initiating LLPS with P54nrb. Structurally, this domain contains IDR region and has been recognized to be involved in dimerization (refer to UniProt database). Hence, developing small molecule inhibitors or antibodies targeting this domain is expected to become a promising therapeutic strategy.

The interplay between vascular and immune microenvironments in cancer treatment has emerged as a promising target. Tumor vascular and immune systems form a complex, interactive network that mutually influences each other deeply [29]. On the one hand, tumor vasculature can secrete immunosuppressive factors such as VEGF to hinder the infiltration and function of immune cells like T cells and tumor‐associated macrophages (TAMs). Additionally, hypoxia within the tumor caused by excessive angiogenesis further exacerbates immunosuppression. On the other hand, immune cells like M2‐polarized TAMs contribute to the formation of a tumor‐supportive vascular system by secreting proangiogenic factors, such as FGF and MMPs. Efficient antiangiogenic therapy is believed to have the potential to alleviate immunosuppression in the microenvironment via remodeling tumor vasculature, thus enhancing the efficacy of immunotherapy. In this study, we expanded our understanding of the microenvironmental characteristics regulated by SRF. We elucidated the vascular microenvironmental changes mediated by SRF and further discovered that these changes exacerbate immunosuppression, primarily manifested by increased infiltration of M2‐polarized macrophages.

There are certain limitations to this study that should be carefully considered. Previous studies have demonstrated that OLFML3 can function as a signaling molecule to directly interact with ECs and immune cells, thereby promoting angiogenesis [30] and immunosuppression [31]. Consequently, a critical question in our ongoing research is to determine whether OLFML3 exerts its effects through ECM degradation, direct action on ECs and immune cells, or both, within the context of SRF's regulation of GBM vascular and immune microenvironment. Additionally, the specific mechanisms underlying the phase separation of the SRF‐P54nrb complex require further elucidation in vitro, and the intricacies of its interaction with the OLFML3 superenhancer warrant further investigation.

## 4 Conclusion

In summary, we focused on the abnormal transcriptional regulatory network involved in the progression of GBM and identified SRF as a key TF that shapes the tumor‐supportive vascular and immune microenvironments. Its elevated expression correlates with higher tumor malignancy and a worse prognosis. Mechanistically, the 133–240aa region of SRF binds to the transcriptional cofactor P54nrb, forming a transcriptional complex that upregulates OLFML3 expression by binding to its enhancer and promoter regions. This process may involve phase separation and super‐enhancer regulation. The secreted glycoprotein OLFML3 activates ECs by degrading and remodeling the ECM, releasing proangiogenic factors and promoting cell–cell adhesion, thereby promoting angiogenesis. The excessive angiogenesis further promotes the activation and infiltration of TAMs, leading to an immunosuppressive microenvironment. In vivo studies revealed that knocking out SRF expression significantly delayed tumor progression and prolonged survival (Figure 7E). Targeting the SRF/P54nrb/OLFML3 axis offers new insights and strategies for improving the prognosis of GBM patients.

## 5 Materials and Methods

### 5.1 Cell Culture and Human Tissue Samples

HUVEC, human brain glial cell line HEB, human glioma cell lines U251, U87MG, A172, T98G, LN229, and human embryonic kidney 293T cells were purchased from the American Type Culture Collection. The U87‐Luc cell line was generated in our laboratory via transfection with a reporter gene encoding firefly luciferase as previously described. All cells were cultured in Dulbecco's modified Eagle's medium (Thermo Scientific, Waltham, MA, USA) supplemented with 10% fetal bovine serum (FBS; Thermo Scientific), 100 IU/mL penicillin G, and 100 µg/mL streptomycin (Invitrogen Life Technologies, Carlsbad, CA, USA). Cells were maintained in a humidified atmosphere containing 5% CO2 at 37°C. All cell lines used in this study obtained certificates within 4 years that authenticated by performing short tandem repeat profiling, and experiments were performed in cells propagated less than 6 months after resuscitation.

A total of 70 cases of formalin‐fixed paraffin‐embedded GBM samples were collected from patients undergoing surgery between 2016 and 2018 at the Department of Pathology, Nanfang Hospital. A total of 10 cases of LGG and GBM were obtained from patients undergoing surgical treatment at Zhujiang Hospital, Southern Medical University. Both study protocol and informed consent were approved by the Ethical Committee of Southern Medical University. The expression of target genes profiling studies on glioma samples were identified through searching in CGGA, TCGA, Rembrandt, Ivy‐GAP, and GEO databases: GSE4412 (*n* = 85), GSE4271 (*n* = 100), GSE53733 (*n* = 70), GSE13041 (*n* = 267), GSE48865 (*n* = 274), GSE16011 (*n* = 284), and GSE82009 (*n* = 50).

### 5.2 RNA Isolation, Reverse Transcription, and Quantitative Real‐Time PCR

Total RNA was extracted using Trizol (Invitrogen, Carlsbad, California). The total RNA was subjected to polyadenylation and reverse transcription (RT) using a ThermoScript RT‐PCR System (Invitrogen). Real‐time polymerase chain reaction (PCR) analysis was carried out using an SYBR Green PCR master mix (Applied Biosystems, Foster City, California) on an ABI 7500HT system. GAPDH was used as an endogenous control. All samples were normalized to internal controls, and fold changes were calculated through relative quantification (2^−ΔΔCT^). The primers used are shown in Table S1.

### 5.3 Luciferase Reporter Assays

OLFML3 was predicted to be the target gene of SRF by using JASPAR software. A 2000‐bp fragment containing two binding sites of the OLFML3 promoter (named WT) was PCR‐amplified and inserted into a pEZX–PL01 luciferase reporter vector. In addition, OLFML3‐binding site mutations (Mutation‐1: Δ(13–30), Mutation‐2: Δ(1660–1677)) were constructed. All the pEZX–PL01 vectors were cotransfected with the SRF‐overexpression vector into 293T and U87MG cells using the lipofectamine 3000 reagent (Thermo Fisher Scientific, USA). Luciferase activity was measured at 48 h after transfection using the Dual‐Luciferase Reporter Assay System (Promega Corporation, Madison, WI, USA).

### 5.4 ChIP and ChIP‐qPCR

After cells were cross‐linked and lysed, DNA fragments were sonicated on ice to lengths between 200 and 1000 bp. Anti‐SRF antibody (1:100; Proteintech) and control immunoglobulin G were used to precipitate protein–DNA complexes. The detail processes were performed according to the instructions of the Pierce Agarose ChIP Kit (Thermo Scientific). Finally, the eluted DNA and 1% of the respective input DNA were reverse cross‐linked at 65°C overnight and used for a PCR assay to examine the putative SRF‐binding sites in the OLFML3 genome with its specific primers (Table S2).

### 5.5 Lentiviral Infection and siRNA Transfection

The lentivirus with SRF oligonucleotides, or SRF sgRNA, P54nrb sgRNA was constructed in Genechem (Shanghai, China). Cells were incubated with lentivirus and polybrene for 24 h. Stable control and specific knockout/overexpressed cells were selected and maintained with puromycin (2 µg/mL; Solarbio, China). The siRNA of OLFML3 and negative control sequences were purchased from Hanbio (Shanghai, China) and transfected using the lipofectamine 3000 reagent (Cat# L3000015; Thermo Fisher Scientific). The sequences of SRF sgRNA are 5′‐CCCAGCTTGGGTCGGTAACA‐3′. The sequences of P54nrb sgRNA are 5′‐GUGUGUCUUCUCUCAGAUGA‐3′. The sequences of OLFML3 siRNA are 5′‐GCUACCAGAUUGUCUAUAATT‐3′.

### 5.6 HUVEC Migration Assay

Transwell assay and wound healing assay were performed to determine the HUVEC migration. For transwell assay, the treated HUVECs were suspended in serum‐free medium and seeded into the transwell chambers with inserts of 8‐µm pore size (BD Biosciences). The medium with 10% FBS was placed into the bottom chamber as a chemoattractant. After 24 h at 37°C, the cells that had migrated through the membrane and stuck to the lower surface of the membrane were stained with hematoxylin and counted under a light microscope in five random visual fields (200×).

### 5.7 Western Blot and Coimmunoprecipitation

RIPA lysis buffer with protease inhibitor cocktail was used to extract total proteins. Proteins were quantified by BCA protein assay kit (Pierce, KeyGEN BioTECH, Jiangsu, China) before separating by SDS‐PAGE gel and transferring onto the PVDF membrane (Millipore, Darmstadt, Germany). Tris buffer containing 0.1% Tween‐20 and 5% nonfat milk was used to block the membrane at 4°C. Rabbit antibodies to SRF (Proteintech; 1:1000), P54nrb (Proteintech; 1:1000), OLFML3 (GeneTex; 1:1000), Flag (Proteintech; 1:1000), mcherry (Proteintech; 1:1000), and GAPDH (1:1000; Proteintech) were used to incubate with the membrane overnight, which is followed by the treatment of HRP‐conjugated secondary antibody (anti‐rabbit IgG/anti‐mouse IgG; CST; 1:10,000). The signal was detected by the enhanced chemiluminescence detection system (Tennon 5200; Shanghai, China) as described by the manufacturer.

Total protein was extracted with NP‐40, incubated with antibody overnight at 4°C, and incubated with protein A/G agarose (Cwbiotech, China) for 3 h. Finally, immunoprecipitates were analyzed via western blot.

### 5.8 GST Pulldown Assay

The GST vectors with or without SRF expression and His–mcherry vectors with or without P54nrb expression were transformed into E. coli strain BL21, and 0.5 mM isopropyl β‐d‐thiogalactoside was added to induce the expression of GST proteins, GST‐SRF fusion proteins, His–mcherry proteins, and His–mcherry–P54nrb fusion proteins at a low temperature of 16°C. Bacteria were lysed by ultrasonic, and purified proteins were harvested by GST‐tag Beaver Beads magnetic beads (BEAVER Life Science, China) and His‐tag Protein Purification Kit (Beyotime, China) based on the manufacturer's instructions. Then, the purified His–mcherry and His–mcherry–P54nrb fusion proteins were coincubated with GST or GST–SRF fusion proteins at 4° overnight. The beads were washed with lysis buffer five times, and the interacting proteins were detected using a western blot assay.

### 5.9 Immunohistochemical and Immunofluorescence Staining

Specimens of surgical tumor tissues from glioma patients and glioma xenograft samples were fixed with 4% formalin, paraffin embedded, and sectioned (4 µm). The tissue sections were then deparaffinized and dehydrated, followed by incubation in 3% hydrogen peroxide for 10 min. Slides were stained with primary antibodies against SRF (Proteintech; 1:400), OLFML3 (GeneTex; 1:400), CD31 (1:100; ZSGB Bio for human samples; 1:200; Abcam for mouse samples), and Ki67 (1:100; ZSGB Bio for human samples; 1:400; Abcam for mouse samples) at 4°C overnight after blocking with 5% BSA in PBS for 1 h at RT. Corresponding secondary antibodies were used for 1 h at RT. Target molecules were detected following DAB staining for immunohistochemistry. Two independent investigators blinded to sample identification, one investigator performed the staining and another one analyzed the glioma tissue section.

For immunofluorescent staining, tissue sections and cells were immunostained with primary antibodies against SRF (Proteintech; 1:400), P54nrb (Proteintech; 1:400), CD163 (1:100; ZSGB Bio for human samples; 1:400; Proteintech for mouse samples), α‐SMA (1:100; ZSGB Bio), and CD31 (1:100; ZSGB Bio for human samples; 1:200; Abcam for mouse samples) overnight at 4°C and subsequently incubated with fluorochrome‐conjugated antibodies. Finally, DAPI was added as a nuclear counterstain. Images were captured using a laser scanning confocal microscope (Nikon, Japan).

Multiplex immunostaining for SRF, OLFML3, CD31, CD163, and α‐SMA was performed using the Opal Polaris 6 Color Automation IHC Detection Kit (Akoya Biosciences, USA). All slides were digitally scanned using a Vectra Polaris scanner (Perkin Elmer, USA), and image analysis was performed using HALOTM (Indica Labs).

### 5.10 Animals and Intracranial Xenograft

Five to eight‐week‐old Balb/c male mice were purchased from the Central Animal Facility of Southern Medical University. The protocols in the study have been approved by the Animal Care and Use Committee of Southern Medical University. U87‐Luc/GL261‐Luc cells were subcutaneously injected stereotactically into the right hemicerebrum of Balb/c nude mice. Each group included five mice. Tumor growth was monitored using an in vivo imaging system (IVIS Lumina II, Caliper, USA) after an intraperitoneal injection of luciferase substrate‐d‐luciferin (YEASEN, Shanghai, China). Mice with neurological deficits or moribund appearance were sacrificed. The tumor‐bearing brains were sectioned for immunohistochemical or immunofluorescence analysis.

### 5.11 Processing and Analysis of Spatial and Single‐Cell Transcriptomics Data

In the context of analyzing spatial and single‐cell transcriptomics data, we employed Seurat as our primary tool for comprehensive visualization and analytical tasks. To address the issue of missing values inherent in scRNA‐seq datasets, we utilized Low‐Rank Approximation of Missing Data for imputation purposes, thereby enhancing dataset completeness [32].

Tumor cells were extracted from the original dataset based on the authors’ annotations and subsequently categorized into two distinct groups: SRFplus (characterized by a median expression value of SRF) and SRFminus (demonstrating an expression level below the median). This segmentation facilitated a more nuanced understanding of cellular heterogeneity within the tumor microenvironment.

Differential gene expression analysis was conducted using the Model‐based Analysis of Single‐cell Transcriptomics, which is tailored for scRNA‐seq data, to identify genes that significantly vary between the SRFplus and SRFminus groups. Additionally, to gain insights into biological pathways enriched among DEGs, we extracted human hallmark gene sets from the Molecular Signatures Database (MSigDB) via the msigdb R package [33]. These gene sets served as the foundation for performing Gene Set Enrichment Analysis (GSEA) through the GSEA R package, thereby elucidating functional implications.

To explore intercellular communication networks, we leveraged the LIANA R package, which specializes in deciphering complex cell–cell communication patterns [34]. Moreover, to uncover transcriptional regulatory activities underlying observed gene expression profiles, we applied the SCENIC algorithm.

For pseudotime trajectory reconstruction, enabling the inference of developmental or differentiation paths within the spatial transcriptomics data, we adopted StLearn, a machine learning‐based approach designed specifically for this purpose. Furthermore, to assess the immune landscape and estimate tumor purity, we utilized the ESTIMATE R package, which provides scores reflective of the immune cell content and overall tumor purity within the sample [35].

### 5.12 Statistical Analysis

The data were analyzed using SPSS version 19.0 software (SPSS, Chicago, IL, USA). The clinical data were analyzed using nonparametric tests (Wilcoxon and Mann–Whitney), Kaplan–Meier, and Cox regression survival analysis. Pearson's chi‐squared (*χ*2) test, unpaired Student's *t*‐test, and paired *t*‐test were used to evaluate the significance of the differences among different groups. All statistical tests were two‐sided. The data are presented as the means ± SD. Figures and graphical elements in this manuscript were created and compiled using BioRender (https://www.biorender.com/ and Adobe Illustrator 2020 adobe, San Jose, CA, USA).

## Author Contributions

Z.‐t.C., Y.‐q.K., L.Z., and F.‐b.Z. conceived and designed the project. Z.‐t.C. and Y.‐j.Z. performed most of the experiments. L.‐w.H. processed and analyzed the spatial and single‐cell transcriptomics data. C.‐y.F. and P.‐q.H. collected and analyzed the MRI data of GBM patients. C.‐y.W., T.‐j.A., J.‐s.J., S.‐s.F., and T.‐l.C. gave assistance in collecting tissue samples and animal experiments. All authors discussed the results and approved the final version.

## Ethics Statement

The formalin‐fixed paraffin‐embedded glioma samples and freshly collected glioma samples were obtained from patients undergoing surgical treatment at Zhujiang Hospital and Nanfang Hospital, Southern Medical University. Both study protocol and informed consent were approved by the Ethical Committee of Southern Medical University (Ethical code: 050432‐4‐2108). Five‐ to eight‐week‐old Balb/c male nude mice were purchased from the Central Animal Facility of Southern Medical University. The protocols in the study have been approved by the Animal Care and Use Committee of Southern Medical University (Application No: NFYY‐2021‐0566). All experiments with nude mice were conducted in accordance with local guidelines on the ethical use of animals and the National Institutes of Health “Guide for the Care and Use of Laboratory Animals.”

## Consent

Consent for publishing has been obtained from the participant.

## Conflicts of Interest

The authors declare no conflicts of interest.

## Supporting information




**Figure S1, related to Figure 1**: (A) Heatmap represents DEGs in indicated datasets. (B) Scatter diagram represents SRF expression level of GBM and LGG tissues in GSE4271, CGGA325, and CGGA693 datasets. (C) Scatter diagram represents SRF expression level of classical, mesenchymal, and proneural subtypes in Ivy‐GAP, GSE48865, and Rembrandt datasets. (D) Scatter diagram represents SRF expression level of human brain tissues and glioma tissues in TCGA, GSE7696, GSE16011, E‐MTAB‐3892, and Rembrandt datasets.
**Figure S2, related to Figure 2**: (A) Heatmap indicated the hierarchical clustering analysis of DEGs between the SRF overexpression and control U87MG cells. GO and pathway enrichment were performed using DEGs. (B) Heatmap indicated the hierarchical clustering analysis of DEGs between the SRF knockout and control G10 cells. GO and pathway enrichment were performed using DEGs. (C) Significantly correlated genes of SRF analyzed in the TCGA–GBM cohort. GO and pathway enrichment were performed using the top 100 correlated genes. (D) RT‐qPCR assay was used to verify the successful construction of SRF overexpression and knock‐out GBM cells. (E) Subcutaneous xenograft tumor model showed the effect of SRF‐overexpression on tumor proliferation. Tumor volume and weight were measured and expressed as mean±SD. (F) Paraffin‐embedded xenograft sections were stained with antibody targeting human CD31. Microvascular density was compared between the SRF.NC and SRF.OE groups. (G) IF assays showed the SRF expression and vessel density of xenograft sections.
**Figure S3, related to Figure 3**: (A) Schematic of the downstream genes (downregulated by SRF) screening process through RNA‐Seq and ChIP‐Seq. (B) The Venn diagram indicated 18 overlapped genes in RNA‐Seq and ChIP‐Seq of SRF. (C) Pearson correlation analysis was conducted to analyze the relation between SRF and OLFML3 in CGGA and TCGA–GBM datasets. (D) SRF expression in WHO grade II, III, and IV gliomas from CGGA301, CGGA325, and CGGA693 datasets. (E) OLFML3 expression level in five regions (cellular tumor, infiltrating tumor, leading edge, microvascular proliferation, and pseudopalisading cells) of GBM tissues from the Ivy‐GAP dataset. (F) Survival curves for GBM patients with low OLFML3 expression versus high OLFML3 expression through analyzing data from CGGA and TCGA glioma databases. (G) Western blot was used to verify the transfect efficacy of OLFML3 overexpression and knockdown in G10 and U87MG cells. (H and I) Representative images of colony formation, transwell, and tube formation assays corresponding to Figure 3G,H. (J) Expression of OLFML3 protein was detected in HUVEC, HA, and seven different GBM cell lines. (K) OLFML3 expression levels across different cell types including tumor cells, ECs, B cells, and so on. Spatial transcriptomics sequencing data were obtained from Gene Expression Omnibus (GSE194329). (L) Effects of OLFML3 knockdown on EC activation were evaluated via tube formation and transwell assays.
**Figure S4, related to Figure 4**: (A) Confocal microscopy images showing the colocalization of SRF and P54nrb in the nucleus of U87MG and G10 cells. (B) Western blot was used to verify the transfect efficacy of P54nrb knockout in G10 and U87MG cells. (C‐E) Representative images of colony formation, transwell and tube formation assay corresponding to Figure 4M–O.
**Figure S5, related to Figure 6**: GO analysis (A) and GSEA (B) results of GL261 RNA‐Seq data.
**Figure S6, related to Figure 7**: (A) Expression levels of SRF and OLFML3 in the SRFplus and SRFminus groups. (B) Dot plot of ligand‐receptor (L–R) pairs of several tumor‐specific pathways between tumor cells (sources) and endothelial cells (receptors), fibroblasts (receptors), oligodendrocytes (receptors), and pericytes (receptors). (C) Dot plot of L–R pairs of several tumor‐specific pathways between tumor cells (receptors) and B cells (sources), granulocytes (sources), myeloid cells (sources), and T cells (sources). (D) Dot plot of L–R pairs of several tumor‐specific pathways between tumor cells (sources) and B cells (receptors), granulocytes (receptors), myeloid cells (receptors), and T cells (receptors).
**Figure S7, related to Figure 7**: (A) Heatmap of the area under the curve (AUC) scores of TF motifs estimated per cell by SCENIC. Shown are differentially activated motifs in SRFplus and SRFminus, respectively. (B) SCENIC analysis predicts TFs such as MAX, THRA, HDAC2, ETV6, IRF2, and JUND as central hubs governing the SRF dysregulation. TF regulatory activities were quantified using AUCell.

**Supplementary Table S1**: RT‐qPCR primer sequences for human genes.
**Supplementary Table S2**: PCR primer sequences.
**Supplementary Table S3**:. univariate and multivariate cox proportional hazards analysis of clinicopathological variables and 24 candidate TFs based on overall survival (OS) in the CGGA325 cohort.
**Supplementary Table S4**: Analysis of clinical parameters associated with SRF expression in CGGA693 cohort.
**Supplementary Table S5**: Peaks information of SRF on the OLFML3 genome.
**Supplementary Table S6**: Potential binding sites of SRF in OLFML3 promoter.
**Supplementary Table S7**: Peaks information of H3K27ac on the OLFML3 genome.
**Supplementary Table S8**: Prediction of phase separation ability of SRF and P54nrb proteins by PhaSepPred. PS‐self score, proteins that can self‐assemble to form condensates. PS‐Part score, proteins whose phase separation behaviors are regulated by protein or nucleic acid partner components. The 8‐feature model incorporates Hydropathy, FCR, IDR, LCR, PScore, PLAAC, catGRANULE, and DeepCoil. The 10‐feature model incorporates the 8 features described above plus Phos frequency and DeepPhase. The ranking of feature values was evaluated in the proteome of the corresponding species. 1‐ranking was shown foreach feature value (The highest Rank score is 1 and the lowest Rank score is 0).

Supporting Information

Supporting Information

Supporting Information

Supporting Information

Supporting Information

Supporting Information

## Data Availability

Data are available upon reasonable request.
