# Supplementary material for: Phase Separation‐Mediated SRF/P54nrb Transcription Complex Shapes the Vasculature Microenvironment via Upregulating OLFML3 in Glioblastoma
Source: MedComm (2020). 2026 May 23;7(6):e70759. doi: 10.1002/mco2.70759 (PMC13239343; doi:10.1002/mco2.70759)
Supplement: Supplementary file 1 — Figure S1, related to Figure 1: (A) Heatmap represents DEGs in indicated datasets. (B) Scatter diagram represents SRF expression level of GBM and LGG tissues in GSE4271, CGGA325, and CGGA693 datasets. (C) Scatter diagram represents SRF expression level of classical, mesenchymal, and proneural subtypes in Ivy‐GAP, GSE48865, and Rembrandt datasets. (D) Scatter diagram represents SRF expression level of human brain tissues and glioma tissues in TCGA, GSE7696, GSE16011, E‐MTAB‐3892, and Rembrandt datasets. Figure S2, related to Figure 2: (A) Heatmap indicated the hierarchical clustering analysis of DEGs between the SRF overexpression and control U87MG cells. GO and pathway enrichment were performed using DEGs. (B) Heatmap indicated the hierarchical clustering analysis of DEGs between the SRF knockout and control G10 cells. GO and pathway enrichment were performed using DEGs. (C) Significantly correlated genes of SRF analyzed in the TCGA–GBM cohort. GO and pathway enrichment were performed using the top 100 correlated genes. (D) RT‐qPCR assay was used to verify the successful construction of SRF overexpression and knock‐out GBM cells. (E) Subcutaneous xenograft tumor model showed the effect of SRF‐overexpression on tumor proliferation. Tumor volume and weight were measured and expressed as mean±SD. (F) Paraffin‐embedded xenograft sections were stained with antibody targeting human CD31. Microvascular density was compared between the SRF.NC and SRF.OE groups. (G) IF assays showed the SRF expression and vessel density of xenograft sections. Figure S3, related to Figure 3: (A) Schematic of the downstream genes (downregulated by SRF) screening process through RNA‐Seq and ChIP‐Seq. (B) The Venn diagram indicated 18 overlapped genes in RNA‐Seq and ChIP‐Seq of SRF. (C) Pearson correlation analysis was conducted to analyze the relation between SRF and OLFML3 in CGGA and TCGA–GBM datasets. (D) SRF expression in WHO grade II, III, and IV gliomas from CGGA301, CGGA325, and CGGA693 [file MCO2-7-e70759-s004.docx]

| **Gene** | **Forward primer** | **Reverse primer** |
| --- | --- | --- |
| SRF | TGATGCTTTTGTGCGAGAAGA | AGGGAAGCGTTTTTATTGGCT |
| OLFML3 | TCCTTTTGTCATGGTCGGGAC | TAAAGCAGCTAGTCGGCGTTC |
| GAPDH | GGAGCGAGATCCCTCCAAAAT | GGCTGTTGTCATACTTCTCATGG |

**Supplementary Table S1.** RT-qPCR primer sequences for human genes.

| **Primer name** | **Forward primer sequence** | **Reverse primer sequence** | **Product length** |
| --- | --- | --- | --- |
| Peak_102450 | GGAGTAAACTGAAGTCTTGAGAATG | AAGAGGAGTTGTGATTAGGAAGG | 110bp |
| Peak_356865 | CATATACCCACCACACAATGATG | TCCAATGATGTAAAGCCAGAAC | 80bp |

**Supplementary Table S2.** PCR primer sequences.

| **OS variables** | **Univariate analysis** | | | | **Multivariate analysis** | | | |
| --- | --- | --- | --- | --- | --- | --- | --- | --- |
|  | **HR** | **L95CI** | **H95CI** | **pvalue** | **HR** | **L95CI** | **H95CI** | **pvalue** |
| **Age** | 1.032862 | 1.020078 | 1.045807 | 3.61E-07 | 1.013728 | 0.998977 | 1.028696 | 0.068285 |
| **Gender** | 0.940529 | 0.715911 | 1.235621 | 0.659668 | - | - | - | - |
| **WHO Grade** | 2.911712 | 2.416675 | 3.508154 | 2.57E-29 | 2.061228 | 1.626447 | 2.612234 | 2.18E-09 |
| **PRS_type** | 1.59726 | 1.368863 | 1.863765 | 2.71E-09 | 1.684962 | 1.397112 | 2.032118 | 4.80E-08 |
| **IDH_mutation** | 0.354499 | 0.268508 | 0.468029 | 2.55E-13 | 0.84572 | 0.505684 | 1.414404 | 0.523071 |
| **1p19q_codeletion** | 0.169786 | 0.104074 | 0.276988 | 1.24E-12 | 0.336724 | 0.18611 | 0.609226 | 0.000321 |
| **MGMTp_methylation** | 0.829505 | 0.632186 | 1.088411 | 0.177432 | - | - | - | - |
| **TEAD3** | 1.128784 | 1.094209 | 1.16445 | 2.31E-14 | 0.886889 | 0.824486 | 0.954015 | 0.001262* |
| **JUNB** | 1.001584 | 1.000739 | 1.002431 | 0.000239 | 0.997536 | 0.995634 | 0.999442 | 0.011298* |
| **SRF** | 1.040508 | 1.027152 | 1.054037 | 1.70E-09 | 1.044299 | 1.008631 | 1.081227 | 0.014498* |
| **ARID5A** | 1.037325 | 1.029227 | 1.045487 | 4.97E-20 | 1.018655 | 1.001705 | 1.035891 | 0.03085* |
| **PKNOX2** | 0.954243 | 0.927782 | 0.981459 | 0.001097 | 0.966388 | 0.934586 | 0.999272 | 0.04522* |
| **HLF** | 0.897709 | 0.874309 | 0.921736 | 1.17E-15 | 0.969766 | 0.930346 | 1.010856 | 0.147056 |
| **TEF** | 0.898135 | 0.87778 | 0.918962 | 4.11E-20 | 0.976606 | 0.939147 | 1.015559 | 0.235518 |
| **ATF3** | 1.003794 | 1.001037 | 1.006558 | 0.006955 | 0.995995 | 0.988747 | 1.003296 | 0.28148 |
| **XBP1** | 1.017558 | 1.01228 | 1.022863 | 5.37E-11 | 1.006105 | 0.994586 | 1.017757 | 0.300208 |
| **ZNF217** | 1.155643 | 1.118036 | 1.194515 | 1.04E-17 | 0.96713 | 0.903518 | 1.035221 | 0.335649 |
| **NCOA2** | 0.88241 | 0.850218 | 0.915821 | 4.18E-11 | 0.969196 | 0.90707 | 1.035577 | 0.354611 |
| **CUX2** | 0.7328 | 0.674271 | 0.796409 | 2.48E-13 | 0.968691 | 0.896572 | 1.04661 | 0.420328 |
| **TGIF1** | 1.039857 | 1.032626 | 1.047139 | 4.95E-28 | 1.005356 | 0.991045 | 1.019874 | 0.465227 |
| **ZNF365** | 0.928085 | 0.899882 | 0.957172 | 2.14E-06 | 1.014974 | 0.972031 | 1.059815 | 0.500401 |
| **PLSCR1** | 1.014535 | 1.010637 | 1.018447 | 2.02E-13 | 0.997891 | 0.990004 | 1.005841 | 0.602021 |
| **KLF10** | 1.033207 | 1.021673 | 1.044871 | 1.17E-08 | 1.006096 | 0.982235 | 1.030537 | 0.619678 |
| **ZNF609** | 0.920485 | 0.888174 | 0.953971 | 5.50E-06 | 1.009952 | 0.954683 | 1.068421 | 0.73018 |
| **NCOA1** | 0.928052 | 0.909716 | 0.946757 | 2.24E-13 | 1.00521 | 0.974159 | 1.03725 | 0.745506 |
| **OLIG2** | 0.996993 | 0.99537 | 0.998618 | 0.000291 | 0.999689 | 0.997679 | 1.001703 | 0.761629 |
| **DBP** | 0.96577 | 0.948654 | 0.983194 | 0.000135 | 0.996717 | 0.975588 | 1.018303 | 0.763535 |
| **SNAI2** | 1.025294 | 1.016021 | 1.034652 | 7.10E-08 | 1.002339 | 0.982911 | 1.022151 | 0.815017 |
| **MAFF** | 1.042579 | 1.027335 | 1.058049 | 2.88E-08 | 0.997261 | 0.970031 | 1.025255 | 0.846029 |
| **THRA** | 0.98816 | 0.985635 | 0.990692 | 7.22E-20 | 1.000188 | 0.995654 | 1.004743 | 0.935312 |
| **ZNF804A** | 0.939088 | 0.865855 | 1.018515 | 0.129243 | - | - | - | - |

**Supplementary Table S3**. univariate and multivariate cox proportional hazards analysis of clinicopathological variables and 24 candidate TFs based on overall survival (OS) in the CGGA325 cohort.

| **Characteristics** | **Total [n]** | **SRF** | |  |
| --- | --- | --- | --- | --- |
|  |  | **Low** | **High** | ***P* value^1^** |
| Age |  |  |  | 0.549028 |
| ≤50 | 505 | 249(49.3) | 256(50.7) |  |
| >50 | 187 | 97（51.9) | 90(48.1) |  |
| Sex |  |  |  | **0.000011** |
| Female | 295 | 141(47.8) | 154(52.2) |  |
| Male | 398 | 205(65.5) | 108(34.5) |  |
| Histology |  |  |  | **1.2314E-71** |
| GBM | 249 | 105(42.2) | 144(57.8) |  |
| A | 119 | 75(63.0) | 44(37.0) |  |
| O | 60 | 31(51.7) | 29(48.3) |  |
| AA | 152 | 62(40.8) | 90(59.2) |  |
| AO | 82 | 46(56.1) | 36(43.9) |  |
| OA | 9 | 8(88.9) | 1(11.1) |  |
| AOA | 21 | 19(90.5) | 2(9.5) |  |
| PRS type |  |  |  | **2.1588E-7** |
| Primary | 422 | 244(57.8) | 178(42.4) |  |
| R and S | 271 | 102(37.6) | 169(62.4) |  |
| Grade |  |  |  | **0.000630** |
| Low | 188 | 114(60.6) | 74(39.4) |  |
| High | 504 | 232(46.0) | 272(54.0) |  |
| WHO grade |  |  |  | **0.000156** |
| I | 0 | 0 | 0 |  |
| II | 188 | 114(60.6) | 74(39.4) |  |
| III | 255 | 127(49.8) | 128(50.2) |  |
| IV | 249 | 105(42.2) | 144(57.8) |  |
| IDH mutation |  |  |  | 0.368968 |
| Wildtype | 286 | 128(44.8) | 158(55.2) |  |
| Mutant | 356 | 172(48.3) | 184(51.7) |  |
| MGMT methylation |  |  |  | 0.992301 |
| Unmethylated | 227 | 119(52.4) | 108(47.6) |  |
| Methylated | 315 | 165(52.4) | 150(47.6) |  |
| 1p19q codeletion |  |  |  | **0.000971** |
| Non-codeletion | 478 | 196(41.0) | 282(59.0) |  |
| Codeletion | 145 | 82(56.6) | 63(43.4) |  |
| Overall survival |  |  |  | **6.4991E-7** |
| Alive | 266 | 165(62.0) | 101(38.0) |  |
| Death | 397 | 168(42.3) | 229(57.7) |  |

**Supplementary Table S4.** Analysis of clinical parameters associated with SRF expression in CGGA693 cohort.

| **Transcript ID** | **Peak name** | **Annotation** | **Start** | **End** | **Int(-10*1og10Pvalue)** |
| --- | --- | --- | --- | --- | --- |
| ENST00000393300.6 | Peak_258087 | Intron | 113971375 | 113971524 | 42 |
| ENST00000633022.1 | Peak_102450 | Intron | 114027897 | 114028258 | 64 |
| ENST00000633022.1 | Peak_356865 | Intron | 114001032 | 114001291 | 29 |
| ENST00000633022.1 | Peak_274901 | Distal Intergenic | 114045998 | 114046147 | 41 |
| ENST00000633022.1 | Peak_99013 | Distal Intergenic | 114043466 | 114043650 | 65 |
| ENST00000633022.1 | Peak_91216 | Distal Intergenic | 114064067 | 114064270 | 66 |

**Supplementary Table S5.** Peaks information of SRF on the OLFML3 genome.

| **Gene** | **Start** | **End** | **Score** | **Strand** | **TFBs** |
| --- | --- | --- | --- | --- | --- |
| OLFML3\|NM_020190.5 | 13 | 30 | 0.830499 | - | cacaaccaaaaaaaggga |
| OLFML3\|NM_020190.5 | 1660 | 1677 | 0.817116 | + | ctttcctaaaacaggaat |
| OLFML3\|NM_020190.5 | 161 | 178 | 0.815676 | + | taatactaactatgggaa |
| OLFML3\|NM_020190.5 | 909 | 926 | 0.81405 | - | atatcccaactaaggctg |
| OLFML3\|NM_020190.5 | 263 | 280 | 0.808621 | + | ttctacataatatggtaa |
| OLFML3\|NM_020190.5 | 265 | 282 | 0.806758 | - | ttttaccatattatgtag |
| OLFML3\|NM_020190.5 | 12 | 29 | 0.801559 | - | acaaccaaaaaaagggag |

**Supplementary Table S6.** Potential binding sites of SRF in OLFML3 promoter.

| **Transcript ID** | **Peak name** | **Annotation** | **Start** | **End** | **Int(-10*1og10Pvalue)** |
| --- | --- | --- | --- | --- | --- |
| ENST00000633022.1 | Peak_21172 | Intron | 114016148 | 114016818 | 61 |
| ENST00000633022.1 | Peak_88178 | Intron | 114025396 | 114026048 | 32 |
| ENST00000633022.1 | Peak_103649 | Exon | 114034892 | 114035335 | 24 |
| ENST00000393300.6 | Peak_50438 | Promoter (≤1kb) | 113978503 | 113978866 | 45 |

**Supplementary Table S7.** Peaks information of H3K27ac on the OLFML3 genome.

|  |
| --- |

|  | **SRF (P11831)** | | | **P54nrb (Q15233)** | |
| --- | --- | --- | --- | --- | --- |
|  | **PS-self score** | **PS-part score** | | **PS-self score** | **PS-part score** |
| Score (8 feature) | 0.674 | 0.202 | | 0.796 | 0.904 |
| Rank (8 feature) | 0.914 | 0.442 | | 0.963 | 0.999 |
| Score (10 feature) | 0.783 | 0.461 | | 0.861 | 0.886 |
| Rank (10 feature) | 0.929 | 0.760 | | 0.963 | 0.967 |
|  | **SRF (P11831)** | | | **P54nrb (Q15233)** | |
|  | **Score** | | **Rank** | **Score** | **Rank** |
| CatGRANULE | 0.981 | | 0.885 | 0.758 | 0.796 |
| PLAAC | 0.028 | | 0.937 | 0.158 | 0.976 |
| Pscore | 3.760 | | 0.938 | 7.53 | 0.995 |
| ESpritz (DisProt) | 0.136 | | 0.761 | 0.183 | 0.804 |
| Hydropathy | 0.472 | | 0.632 | 0.389 | 0.045 |
| SEG | 0.287 | | 0.937 | 0.261 | 0.922 |
| Charged residue | 0.140 | | 0.082 | 0.299 | 0.896 |
| Phos frequency | 0.045 | | 0.901 | 0.047 | 0.907 |
| DeepPhase | 0.384 | | 0.197 | 0.393 | 0.213 |
| DeepCoil | 0.000 | | / | 0.000 | / |

**Supplementary Table S8.** Prediction of phase separation ability of SRF and P54nrb proteins by PhaSepPred. PS-self score, proteins that can self-assemble to form condensates. PS-Part score, proteins whose phase separation behaviors are regulated by protein or nucleic acid partner components. The 8-feature model incorporates Hydropathy, FCR, IDR, LCR, PScore, PLAAC, catGRANULE, and DeepCoil. The 10-feature model incorporates the 8 features described above plus Phos frequency and DeepPhase. The ranking of feature values was evaluated in the proteome of the corresponding species. 1-ranking was shown foreach feature value (The highest Rank score is 1 and the lowest Rank score is 0).

**Figure S1.**


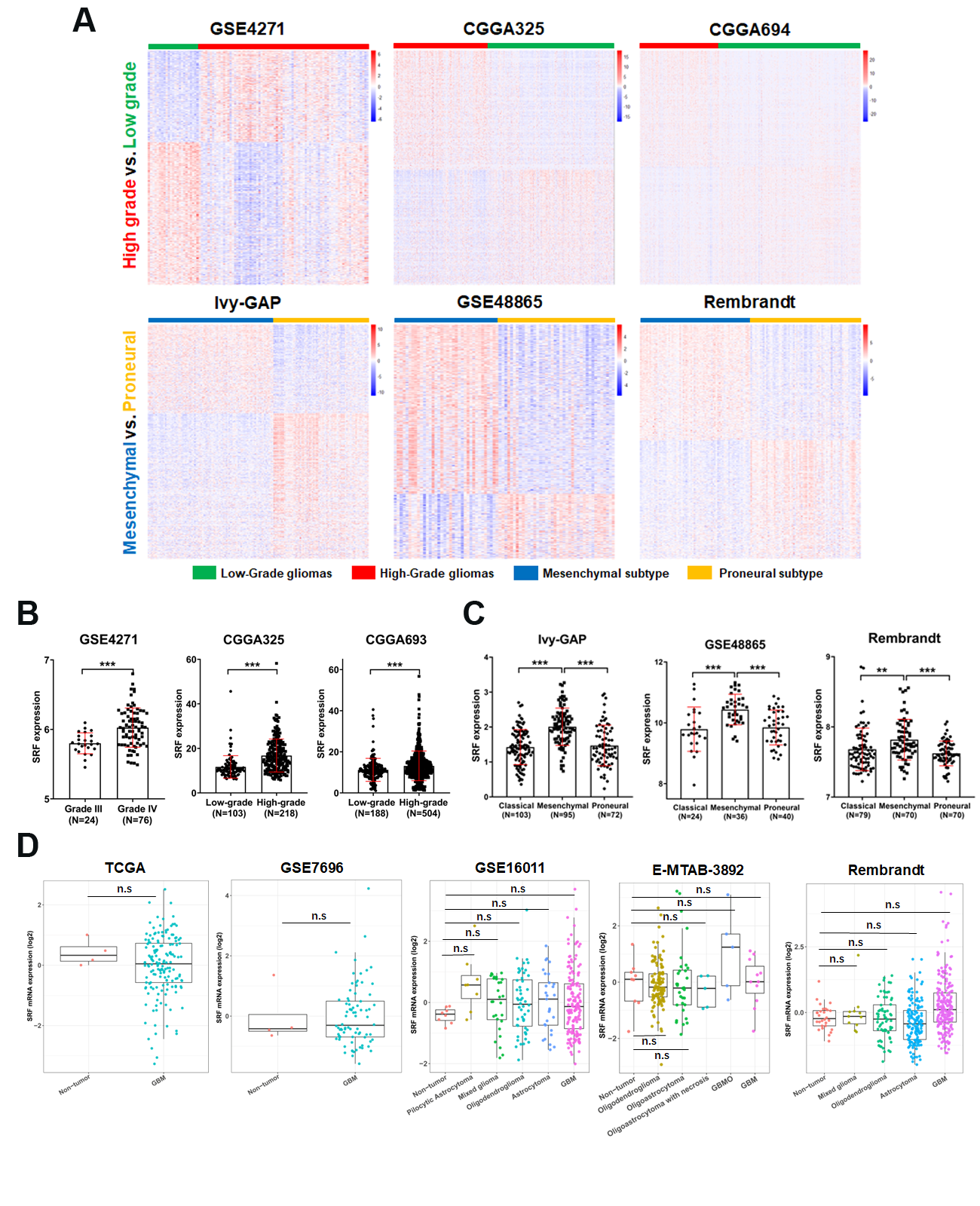


**Figure S1, related to Figure 1.** (A) Heatmap represents DEGs in indicated datasets. (B) Scatter diagram represents SRF expression level of GBM and LGG tissues in GSE4271, CGGA325 and CGGA693 datasets. (C) Scatter diagram represents SRF expression level of classical, mesenchymal, and proneural subtypes in Ivy-GAP, GSE48865, and Rembrandt datasets. (D) Scatter diagram represents SRF expression level of human brain tissues and glioma tissues in TCGA, GSE7696, GSE16011, E-MTAB-3892, and Rembrandt datasets.

**Figure S2.**


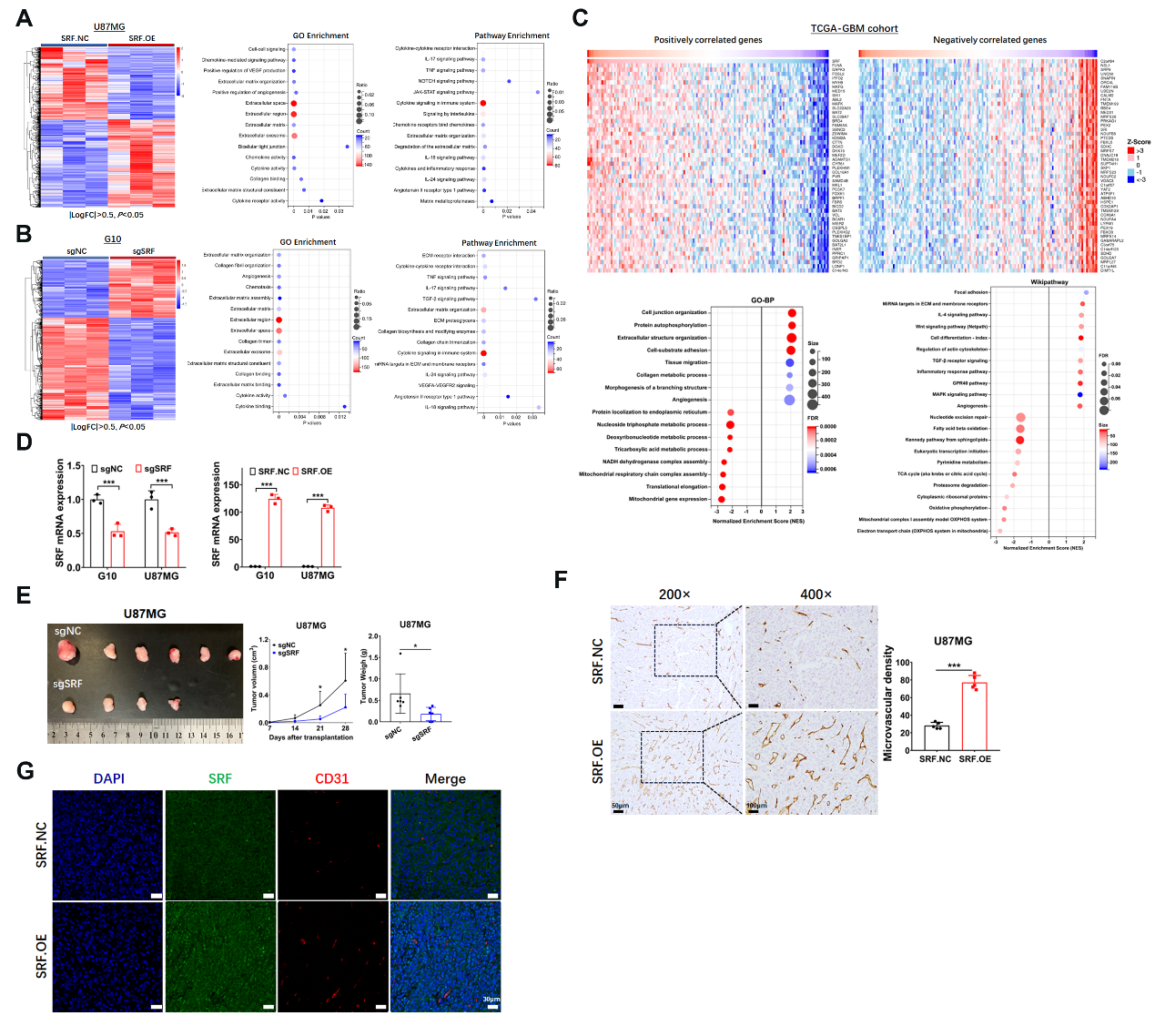


**Figure S2, related to Figure 2.** (A) Heatmap indicated the hierarchical clustering analysis of DEGs between the SRF overexpression and control U87MG cells. GO and pathway enrichment were performed using DEGs. (B) Heatmap indicated the hierarchical clustering analysis of DEGs between the SRF knockout and control G10 cells. GO and pathway enrichment were performed using DEGs. (C) Significantly correlated genes of SRF analyzed in the TCGA-GBM cohort. GO and pathway enrichment were performed using the top 100 correlated genes. (D) RT-qPCR assay was used to verify the successful construction of SRF overexpression and knock-out GBM cells. (E) Subcutaneous xenograft tumor model showed the effect of SRF-overexpression on tumor proliferation. Tumor volume and weight were measured and expressed as mean±SD. (F) Paraffin-embedded xenograft sections were stained with antibody targeting human CD31. Microvascular density was compared between the SRF.NC and SRF.OE groups. (G) IF assays showed the SRF expression and vessel density of xenograft sections.

**Figure S3.**


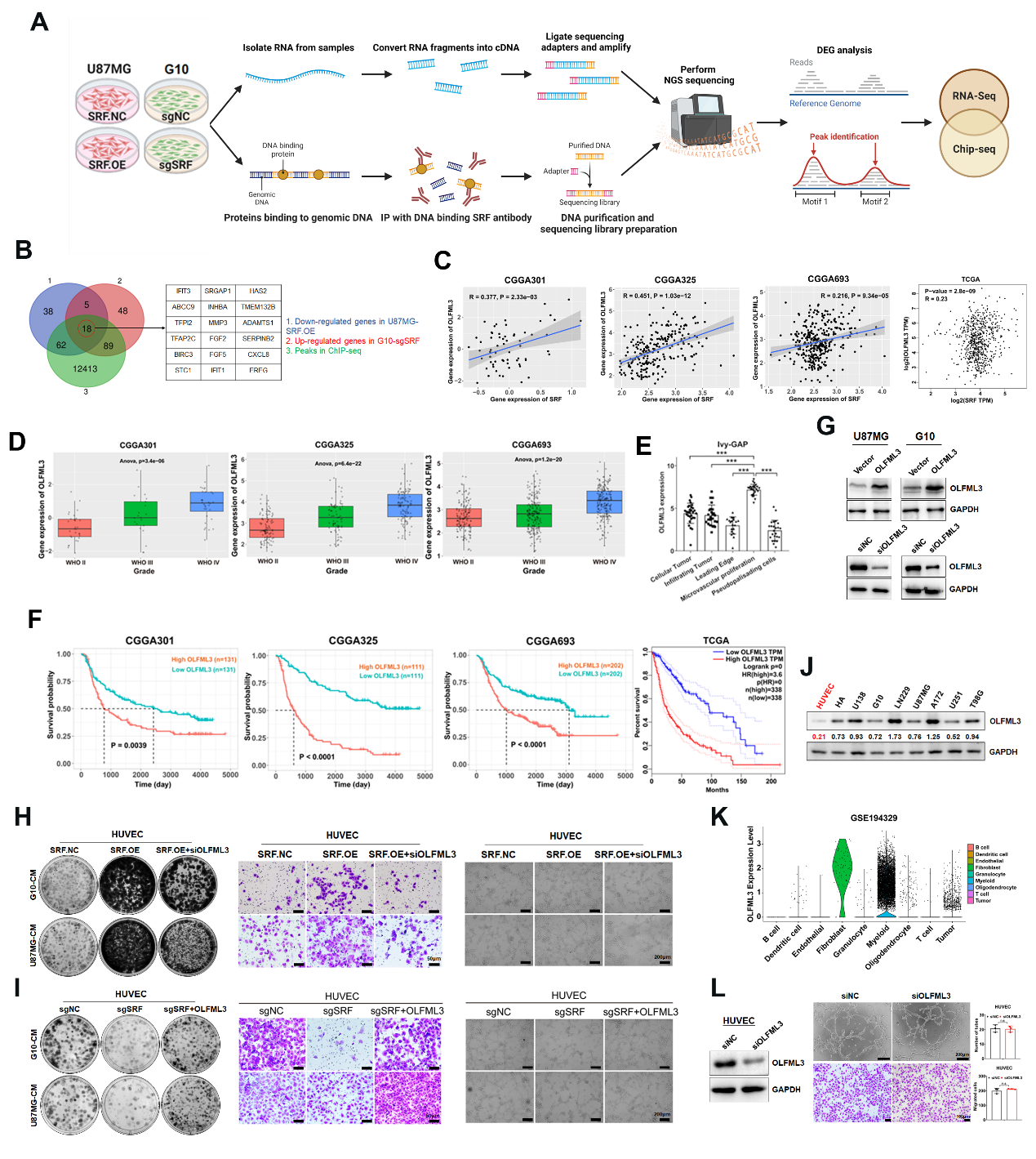


**Figure S3, related to Figure 3.** (A) Schematic of the downstream genes (downregulated by SRF) screening process through RNA-Seq and ChIP-Seq. (B) The Venn diagram indicated 18 overlapped genes in RNA-Seq and ChIP-Seq of SRF. (C) Pearson correlation analysis was conducted to analyze the relation between SRF and OLFML3 in CGGA and TCGA-GBM datasets. (D) SRF expression in WHO grade II, III, and IV gliomas from CGGA301, CGGA325 and CGGA693 datasets. (E) OLFML3 expression level in 5 regions (cellular tumor, infiltrating tumor, leading edge, microvascular proliferation, and pseudopalisading cells) of GBM tissues from the Ivy-GAP dataset. (F) Survival curves for GBM patients with low OLFML3 expression versus high OLFML3 expression through analyzing data from CGGA and TCGA glioma databases. (G) Western blot was used to verify the transfect efficacy of OLFML3 overexpression and knockdown in G10 and U87MG cells. (H and I) Representative images of colony formation, transwell, and tube formation assays corresponding to Figures 3G and 3H. (J) Expression of OLFML3 protein was detected in HUVEC, HA and 7 different GBM cell lines. (K) OLFML3 expression levels across different cell types including tumor cells, ECs, B cells, etc. Spatial transcriptomics sequencing data was obtained from Gene Expression Omnibus (GSE194329). (L) Effects of OLFML3 knockdown on EC activation were evaluated via tube formation and transwell assays.

**Figure S4.**


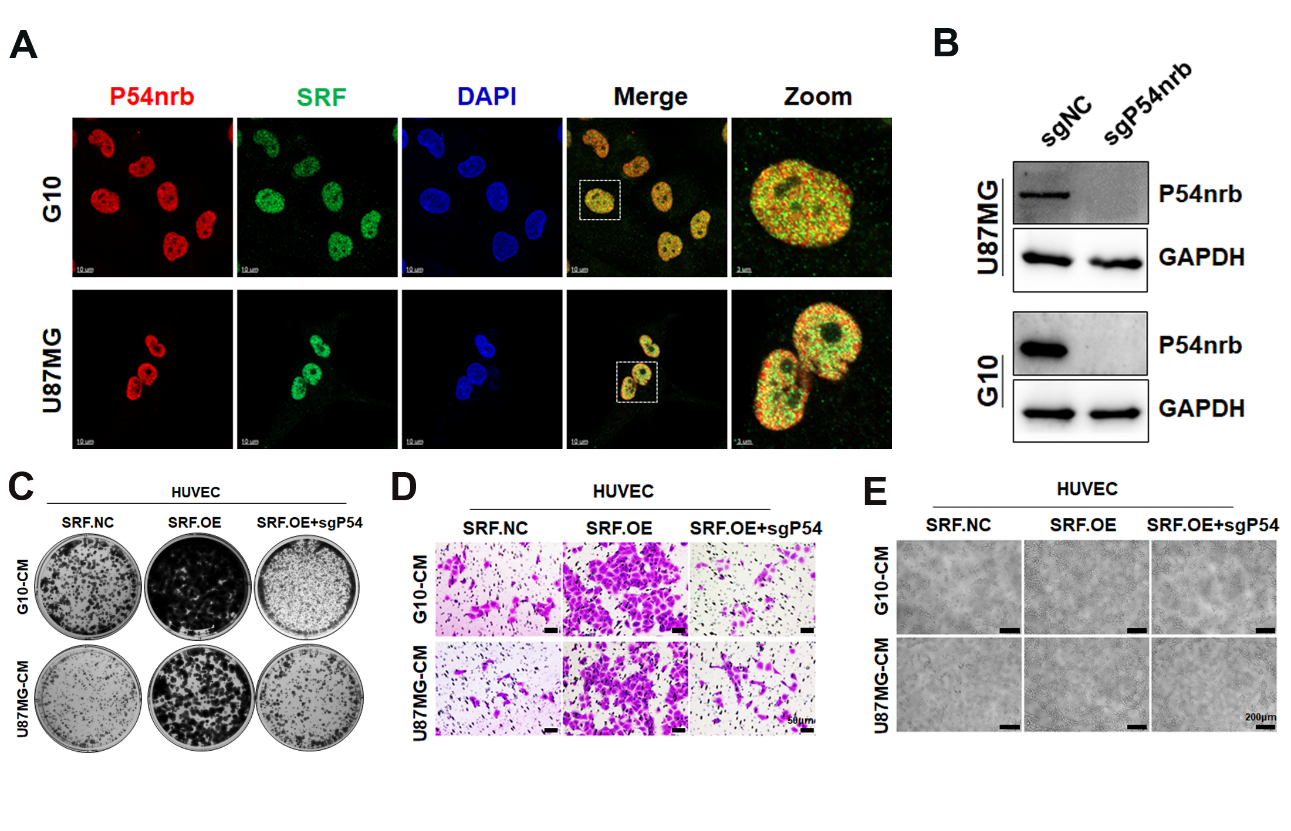


**Figure S4, related to Figure 4.** (A) Confocal microscopy images showing the colocalization of SRF and P54nrb in the nucleus of U87MG and G10 cells. (B) Western blot was used to verify the transfect efficacy of P54nrb knockout in G10 and U87MG cells. (C-E) Representative images of colony formation, transwell and tube formation assay corresponding to Figure 4M-O.

**Figure S5.**


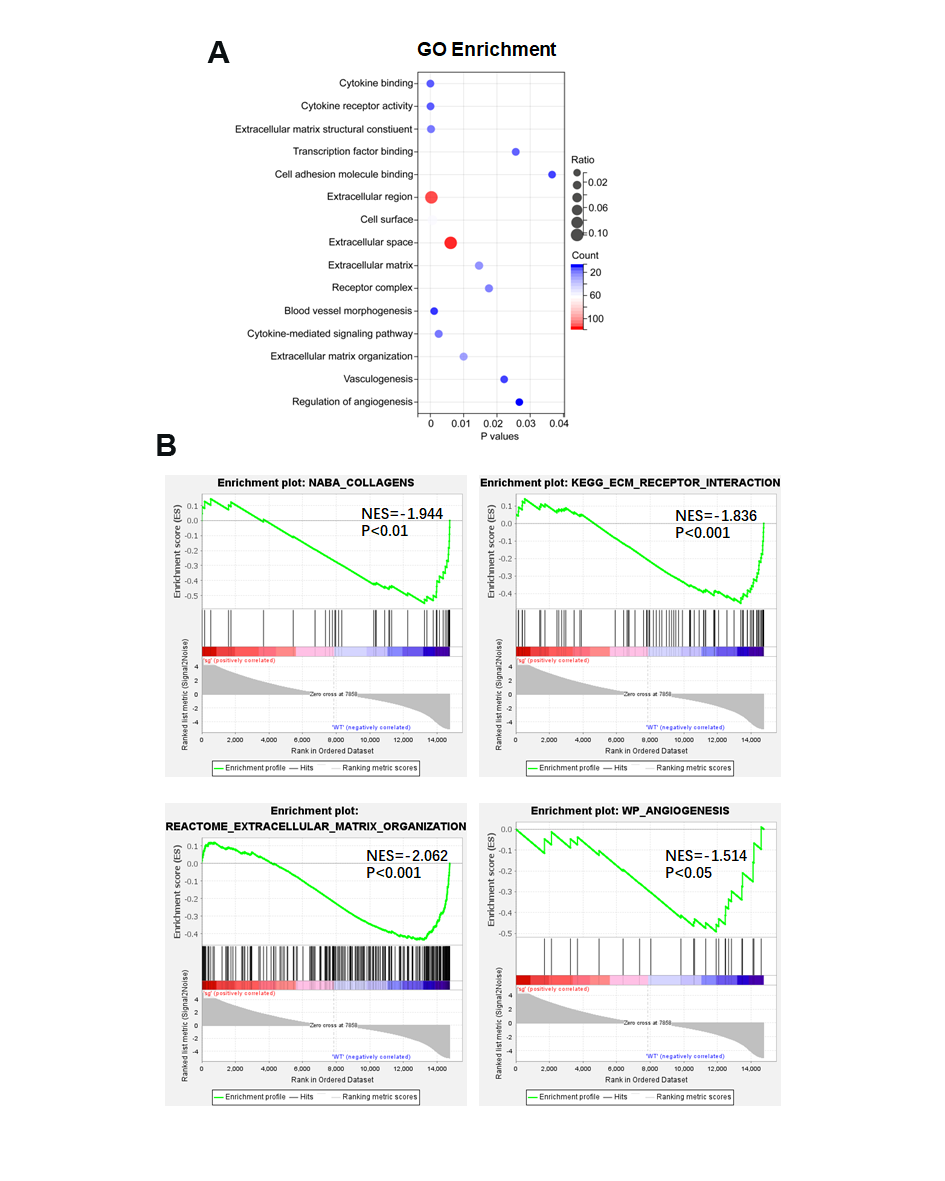


**Figure S5, related to Figure 6.** GO analysis (A) and GSEA (B) results of GL261 RNA-Seq data.

**Figure S6.**


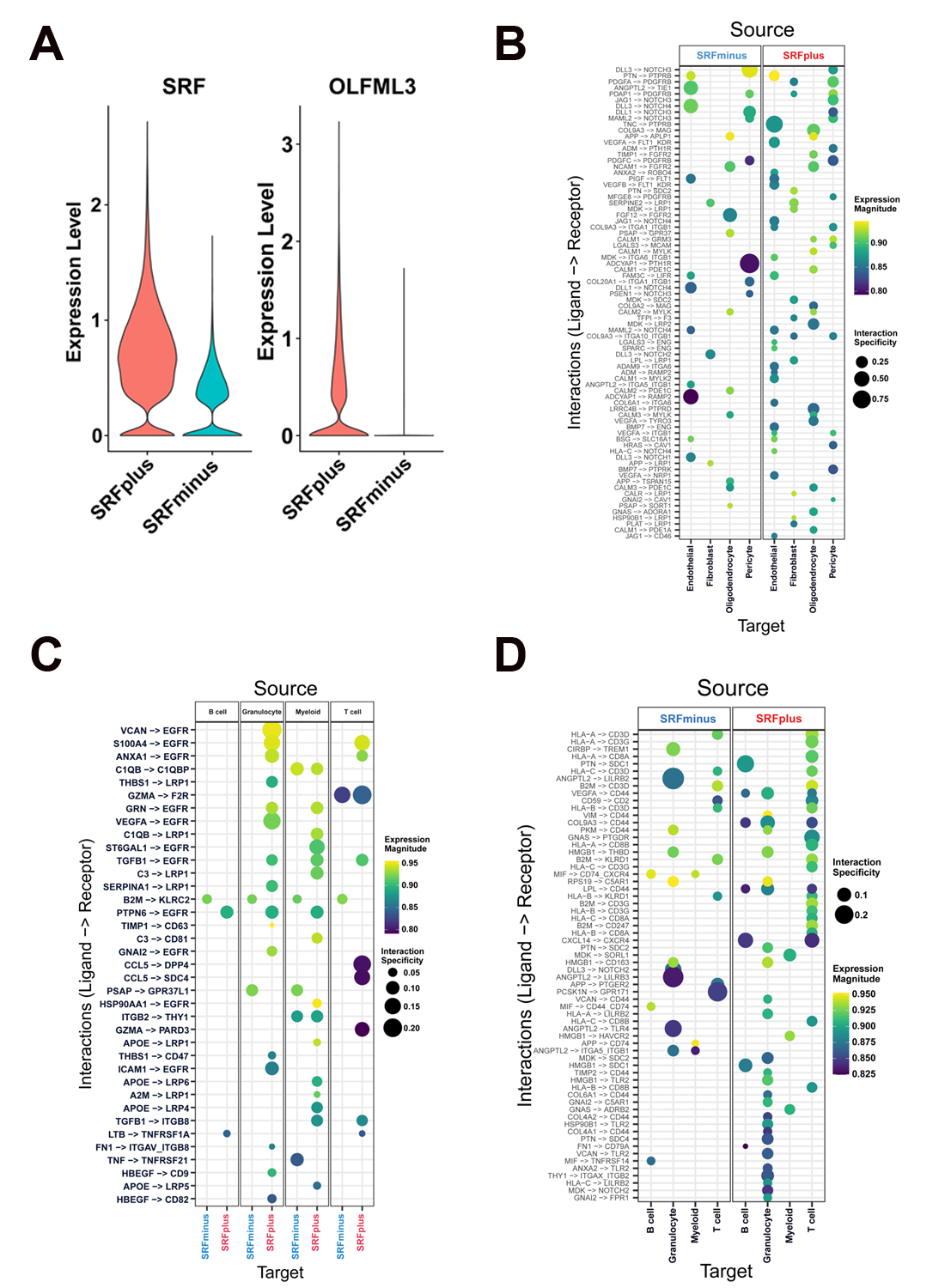


**Figure S6, related to Figure 7.** (A) Expression levels of SRF and OLFML3 in the SRFplus and SRFminus groups. (B) Dot plot of ligand-receptor (L-R) pairs of several tumor-specific pathways between tumor cells (sources) and endothelial cells (receptors), fibroblasts (receptors), oligodendrocytes (receptors), and pericytes (receptors). (C) Dot plot of L-R pairs of several tumor-specific pathways between tumor cells (receptors) and B cells (sources), granulocytes (sources), myeloid cells (sources), and T cells (sources). (D) Dot plot of L-R pairs of several tumor-specific pathways between tumor cells (sources) and B cells (receptors), granulocytes (receptors), myeloid cells (receptors), and T cells (receptors).

**Figure S7.
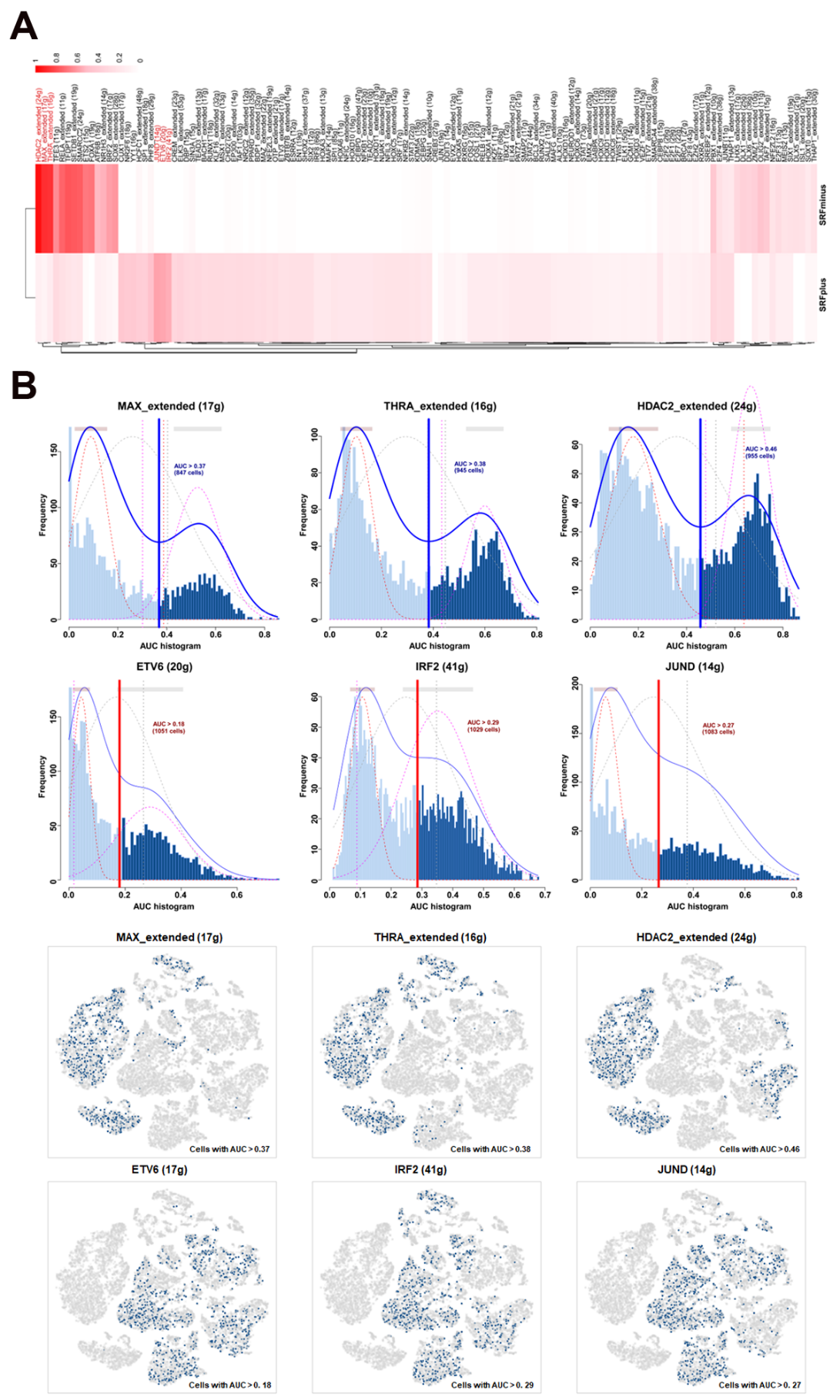
**

**Figure S7, related to Figure 7.** (A) Heatmap of the area under the curve (AUC) scores of TF motifs estimated per cell by SCENIC. Shown are differentially activated motifs in SRFplus and SRFminus, respectively. (B) SCENIC analysis predicts TFs such as MAX, THRA, HDAC2, ETV6, IRF2, and JUND as central hubs governing the SRF dysregulation. TF regulatory activities were quantified using AUCell.
