# Supplementary material for: Phase Separation‐Mediated SRF/P54nrb Transcription Complex Shapes the Vasculature Microenvironment via Upregulating OLFML3 in Glioblastoma
Source: MedComm (2020). 2026 May 23;7(6):e70759. doi: 10.1002/mco2.70759 (PMC13239343; doi:10.1002/mco2.70759)
Supplement: Supplementary file 2 — Supplementary Table S1: RT‐qPCR primer sequences for human genes. Supplementary Table S2: PCR primer sequences. Supplementary Table S3:. univariate and multivariate cox proportional hazards analysis of clinicopathological variables and 24 candidate TFs based on overall survival (OS) in the CGGA325 cohort. Supplementary Table S4: Analysis of clinical parameters associated with SRF expression in CGGA693 cohort. Supplementary Table S5: Peaks information of SRF on the OLFML3 genome. Supplementary Table S6: Potential binding sites of SRF in OLFML3 promoter. Supplementary Table S7: Peaks information of H3K27ac on the OLFML3 genome. Supplementary Table S8: Prediction of phase separation ability of SRF and P54nrb proteins by PhaSepPred. PS‐self score, proteins that can self‐assemble to form condensates. PS‐Part score, proteins whose phase separation behaviors are regulated by protein or nucleic acid partner components. The 8‐feature model incorporates Hydropathy, FCR, IDR, LCR, PScore, PLAAC, catGRANULE, and DeepCoil. The 10‐feature model incorporates the 8 features described above plus Phos frequency and DeepPhase. The ranking of feature values was evaluated in the proteome of the corresponding species. 1‐ranking was shown foreach feature value (The highest Rank score is 1 and the lowest Rank score is 0). [file MCO2-7-e70759-s008.docx]

| **Gene** | **Forward primer** | **Reverse primer** |
| --- | --- | --- |
| SRF | TGATGCTTTTGTGCGAGAAGA | AGGGAAGCGTTTTTATTGGCT |
| OLFML3 | TCCTTTTGTCATGGTCGGGAC | TAAAGCAGCTAGTCGGCGTTC |
| GAPDH | GGAGCGAGATCCCTCCAAAAT | GGCTGTTGTCATACTTCTCATGG |

**Supplementary Table S1.** RT-qPCR primer sequences for human genes.

| **Primer name** | **Forward primer sequence** | **Reverse primer sequence** | **Product length** |
| --- | --- | --- | --- |
| Peak_102450 | GGAGTAAACTGAAGTCTTGAGAATG | AAGAGGAGTTGTGATTAGGAAGG | 110bp |
| Peak_356865 | CATATACCCACCACACAATGATG | TCCAATGATGTAAAGCCAGAAC | 80bp |

**Supplementary Table S2.** PCR primer sequences.

| **OS variables** | **Univariate analysis** | | | | **Multivariate analysis** | | | |
| --- | --- | --- | --- | --- | --- | --- | --- | --- |
|  | **HR** | **L95CI** | **H95CI** | **pvalue** | **HR** | **L95CI** | **H95CI** | **pvalue** |
| **Age** | 1.032862 | 1.020078 | 1.045807 | 3.61E-07 | 1.013728 | 0.998977 | 1.028696 | 0.068285 |
| **Gender** | 0.940529 | 0.715911 | 1.235621 | 0.659668 | - | - | - | - |
| **WHO Grade** | 2.911712 | 2.416675 | 3.508154 | 2.57E-29 | 2.061228 | 1.626447 | 2.612234 | 2.18E-09 |
| **PRS_type** | 1.59726 | 1.368863 | 1.863765 | 2.71E-09 | 1.684962 | 1.397112 | 2.032118 | 4.80E-08 |
| **IDH_mutation** | 0.354499 | 0.268508 | 0.468029 | 2.55E-13 | 0.84572 | 0.505684 | 1.414404 | 0.523071 |
| **1p19q_codeletion** | 0.169786 | 0.104074 | 0.276988 | 1.24E-12 | 0.336724 | 0.18611 | 0.609226 | 0.000321 |
| **MGMTp_methylation** | 0.829505 | 0.632186 | 1.088411 | 0.177432 | - | - | - | - |
| **TEAD3** | 1.128784 | 1.094209 | 1.16445 | 2.31E-14 | 0.886889 | 0.824486 | 0.954015 | 0.001262* |
| **JUNB** | 1.001584 | 1.000739 | 1.002431 | 0.000239 | 0.997536 | 0.995634 | 0.999442 | 0.011298* |
| **SRF** | 1.040508 | 1.027152 | 1.054037 | 1.70E-09 | 1.044299 | 1.008631 | 1.081227 | 0.014498* |
| **ARID5A** | 1.037325 | 1.029227 | 1.045487 | 4.97E-20 | 1.018655 | 1.001705 | 1.035891 | 0.03085* |
| **PKNOX2** | 0.954243 | 0.927782 | 0.981459 | 0.001097 | 0.966388 | 0.934586 | 0.999272 | 0.04522* |
| **HLF** | 0.897709 | 0.874309 | 0.921736 | 1.17E-15 | 0.969766 | 0.930346 | 1.010856 | 0.147056 |
| **TEF** | 0.898135 | 0.87778 | 0.918962 | 4.11E-20 | 0.976606 | 0.939147 | 1.015559 | 0.235518 |
| **ATF3** | 1.003794 | 1.001037 | 1.006558 | 0.006955 | 0.995995 | 0.988747 | 1.003296 | 0.28148 |
| **XBP1** | 1.017558 | 1.01228 | 1.022863 | 5.37E-11 | 1.006105 | 0.994586 | 1.017757 | 0.300208 |
| **ZNF217** | 1.155643 | 1.118036 | 1.194515 | 1.04E-17 | 0.96713 | 0.903518 | 1.035221 | 0.335649 |
| **NCOA2** | 0.88241 | 0.850218 | 0.915821 | 4.18E-11 | 0.969196 | 0.90707 | 1.035577 | 0.354611 |
| **CUX2** | 0.7328 | 0.674271 | 0.796409 | 2.48E-13 | 0.968691 | 0.896572 | 1.04661 | 0.420328 |
| **TGIF1** | 1.039857 | 1.032626 | 1.047139 | 4.95E-28 | 1.005356 | 0.991045 | 1.019874 | 0.465227 |
| **ZNF365** | 0.928085 | 0.899882 | 0.957172 | 2.14E-06 | 1.014974 | 0.972031 | 1.059815 | 0.500401 |
| **PLSCR1** | 1.014535 | 1.010637 | 1.018447 | 2.02E-13 | 0.997891 | 0.990004 | 1.005841 | 0.602021 |
| **KLF10** | 1.033207 | 1.021673 | 1.044871 | 1.17E-08 | 1.006096 | 0.982235 | 1.030537 | 0.619678 |
| **ZNF609** | 0.920485 | 0.888174 | 0.953971 | 5.50E-06 | 1.009952 | 0.954683 | 1.068421 | 0.73018 |
| **NCOA1** | 0.928052 | 0.909716 | 0.946757 | 2.24E-13 | 1.00521 | 0.974159 | 1.03725 | 0.745506 |
| **OLIG2** | 0.996993 | 0.99537 | 0.998618 | 0.000291 | 0.999689 | 0.997679 | 1.001703 | 0.761629 |
| **DBP** | 0.96577 | 0.948654 | 0.983194 | 0.000135 | 0.996717 | 0.975588 | 1.018303 | 0.763535 |
| **SNAI2** | 1.025294 | 1.016021 | 1.034652 | 7.10E-08 | 1.002339 | 0.982911 | 1.022151 | 0.815017 |
| **MAFF** | 1.042579 | 1.027335 | 1.058049 | 2.88E-08 | 0.997261 | 0.970031 | 1.025255 | 0.846029 |
| **THRA** | 0.98816 | 0.985635 | 0.990692 | 7.22E-20 | 1.000188 | 0.995654 | 1.004743 | 0.935312 |
| **ZNF804A** | 0.939088 | 0.865855 | 1.018515 | 0.129243 | - | - | - | - |

**Supplementary Table S3**. univariate and multivariate cox proportional hazards analysis of clinicopathological variables and 24 candidate TFs based on overall survival (OS) in the CGGA325 cohort.

| **Characteristics** | **Total [n]** | **SRF** | |  |
| --- | --- | --- | --- | --- |
|  |  | **Low** | **High** | ***P* value^1^** |
| Age |  |  |  | 0.549028 |
| ≤50 | 505 | 249(49.3) | 256(50.7) |  |
| >50 | 187 | 97（51.9) | 90(48.1) |  |
| Sex |  |  |  | **0.000011** |
| Female | 295 | 141(47.8) | 154(52.2) |  |
| Male | 398 | 205(65.5) | 108(34.5) |  |
| Histology |  |  |  | **1.2314E-71** |
| GBM | 249 | 105(42.2) | 144(57.8) |  |
| A | 119 | 75(63.0) | 44(37.0) |  |
| O | 60 | 31(51.7) | 29(48.3) |  |
| AA | 152 | 62(40.8) | 90(59.2) |  |
| AO | 82 | 46(56.1) | 36(43.9) |  |
| OA | 9 | 8(88.9) | 1(11.1) |  |
| AOA | 21 | 19(90.5) | 2(9.5) |  |
| PRS type |  |  |  | **2.1588E-7** |
| Primary | 422 | 244(57.8) | 178(42.4) |  |
| R and S | 271 | 102(37.6) | 169(62.4) |  |
| Grade |  |  |  | **0.000630** |
| Low | 188 | 114(60.6) | 74(39.4) |  |
| High | 504 | 232(46.0) | 272(54.0) |  |
| WHO grade |  |  |  | **0.000156** |
| I | 0 | 0 | 0 |  |
| II | 188 | 114(60.6) | 74(39.4) |  |
| III | 255 | 127(49.8) | 128(50.2) |  |
| IV | 249 | 105(42.2) | 144(57.8) |  |
| IDH mutation |  |  |  | 0.368968 |
| Wildtype | 286 | 128(44.8) | 158(55.2) |  |
| Mutant | 356 | 172(48.3) | 184(51.7) |  |
| MGMT methylation |  |  |  | 0.992301 |
| Unmethylated | 227 | 119(52.4) | 108(47.6) |  |
| Methylated | 315 | 165(52.4) | 150(47.6) |  |
| 1p19q codeletion |  |  |  | **0.000971** |
| Non-codeletion | 478 | 196(41.0) | 282(59.0) |  |
| Codeletion | 145 | 82(56.6) | 63(43.4) |  |
| Overall survival |  |  |  | **6.4991E-7** |
| Alive | 266 | 165(62.0) | 101(38.0) |  |
| Death | 397 | 168(42.3) | 229(57.7) |  |

**Supplementary Table S4.** Analysis of clinical parameters associated with SRF expression in CGGA693 cohort.

| **Transcript ID** | **Peak name** | **Annotation** | **Start** | **End** | **Int(-10*1og10Pvalue)** |
| --- | --- | --- | --- | --- | --- |
| ENST00000393300.6 | Peak_258087 | Intron | 113971375 | 113971524 | 42 |
| ENST00000633022.1 | Peak_102450 | Intron | 114027897 | 114028258 | 64 |
| ENST00000633022.1 | Peak_356865 | Intron | 114001032 | 114001291 | 29 |
| ENST00000633022.1 | Peak_274901 | Distal Intergenic | 114045998 | 114046147 | 41 |
| ENST00000633022.1 | Peak_99013 | Distal Intergenic | 114043466 | 114043650 | 65 |
| ENST00000633022.1 | Peak_91216 | Distal Intergenic | 114064067 | 114064270 | 66 |

**Supplementary Table S5.** Peaks information of SRF on the OLFML3 genome.

| **Gene** | **Start** | **End** | **Score** | **Strand** | **TFBs** |
| --- | --- | --- | --- | --- | --- |
| OLFML3\|NM_020190.5 | 13 | 30 | 0.830499 | - | cacaaccaaaaaaaggga |
| OLFML3\|NM_020190.5 | 1660 | 1677 | 0.817116 | + | ctttcctaaaacaggaat |
| OLFML3\|NM_020190.5 | 161 | 178 | 0.815676 | + | taatactaactatgggaa |
| OLFML3\|NM_020190.5 | 909 | 926 | 0.81405 | - | atatcccaactaaggctg |
| OLFML3\|NM_020190.5 | 263 | 280 | 0.808621 | + | ttctacataatatggtaa |
| OLFML3\|NM_020190.5 | 265 | 282 | 0.806758 | - | ttttaccatattatgtag |
| OLFML3\|NM_020190.5 | 12 | 29 | 0.801559 | - | acaaccaaaaaaagggag |

**Supplementary Table S6.** Potential binding sites of SRF in OLFML3 promoter.

| **Transcript ID** | **Peak name** | **Annotation** | **Start** | **End** | **Int(-10*1og10Pvalue)** |
| --- | --- | --- | --- | --- | --- |
| ENST00000633022.1 | Peak_21172 | Intron | 114016148 | 114016818 | 61 |
| ENST00000633022.1 | Peak_88178 | Intron | 114025396 | 114026048 | 32 |
| ENST00000633022.1 | Peak_103649 | Exon | 114034892 | 114035335 | 24 |
| ENST00000393300.6 | Peak_50438 | Promoter (≤1kb) | 113978503 | 113978866 | 45 |

**Supplementary Table S7.** Peaks information of H3K27ac on the OLFML3 genome.

|  |
| --- |

|  | **SRF (P11831)** | | | **P54nrb (Q15233)** | |
| --- | --- | --- | --- | --- | --- |
|  | **PS-self score** | **PS-part score** | | **PS-self score** | **PS-part score** |
| Score (8 feature) | 0.674 | 0.202 | | 0.796 | 0.904 |
| Rank (8 feature) | 0.914 | 0.442 | | 0.963 | 0.999 |
| Score (10 feature) | 0.783 | 0.461 | | 0.861 | 0.886 |
| Rank (10 feature) | 0.929 | 0.760 | | 0.963 | 0.967 |
|  | **SRF (P11831)** | | | **P54nrb (Q15233)** | |
|  | **Score** | | **Rank** | **Score** | **Rank** |
| CatGRANULE | 0.981 | | 0.885 | 0.758 | 0.796 |
| PLAAC | 0.028 | | 0.937 | 0.158 | 0.976 |
| Pscore | 3.760 | | 0.938 | 7.53 | 0.995 |
| ESpritz (DisProt) | 0.136 | | 0.761 | 0.183 | 0.804 |
| Hydropathy | 0.472 | | 0.632 | 0.389 | 0.045 |
| SEG | 0.287 | | 0.937 | 0.261 | 0.922 |
| Charged residue | 0.140 | | 0.082 | 0.299 | 0.896 |
| Phos frequency | 0.045 | | 0.901 | 0.047 | 0.907 |
| DeepPhase | 0.384 | | 0.197 | 0.393 | 0.213 |
| DeepCoil | 0.000 | | / | 0.000 | / |

**Supplementary Table S8.** Prediction of phase separation ability of SRF and P54nrb proteins by PhaSepPred. PS-self score, proteins that can self-assemble to form condensates. PS-Part score, proteins whose phase separation behaviors are regulated by protein or nucleic acid partner components. The 8-feature model incorporates Hydropathy, FCR, IDR, LCR, PScore, PLAAC, catGRANULE, and DeepCoil. The 10-feature model incorporates the 8 features described above plus Phos frequency and DeepPhase. The ranking of feature values was evaluated in the proteome of the corresponding species. 1-ranking was shown foreach feature value (The highest Rank score is 1 and the lowest Rank score is 0).
